# Progress in Nickel MOF-Based Materials for Electrochemical Biosensor and Supercapacitor Applications

**DOI:** 10.3390/bios15090560

**Published:** 2025-08-25

**Authors:** Shanmugam Vignesh, Khursheed Ahmad, Tae Hwan Oh

**Affiliations:** School of Chemical Engineering, Yeungnam University, 280 Daehak-Ro, Gyeongsan 38541, Republic of Korea; vigneshattur1@yu.ac.kr

**Keywords:** Ni-MOF, electrochemical sensors, biosensors, environmental pollutants, supercapacitors, biomolecules

## Abstract

Nickel-based metal–organic frameworks (Ni-MOFs) have received enormous amounts of attention from the scientific community due to their excellent porosity, larger specific surface area, tunable structure, and intrinsic redox properties. In previous years, Ni-MOFs and their hybrid composite materials have been extensively explored for electrochemical sensing applications. As per the reported literature, Ni-MOF-based hybrid materials have been used in the fabrication of electrochemical sensors for the monitoring of ascorbic acid, glucose, L-tryptophan, bisphenol A, carbendazim, catechol, hydroquinone, 4-chlorophenol, uric acid, kaempferol, adenine, _L_-cysteine, etc. The presence of synergistic effects in Ni-MOF-based hybrid materials plays a crucial role in the development of highly selective electrochemical sensors. Thus, Ni-MOF-based materials exhibited enhanced sensitivity and selectivity with reasonable real sample recovery, which suggested their potential for practical applications. In addition, Ni-MOF-based hybrid composites were also adopted as electrode modifiers for the development of supercapacitors. The Ni-MOF-based materials demonstrated excellent specific capacitance at low current densities with reasonable cyclic stability. This review article provides an overview of recent advancements in the utilization of Ni-MOF-based electrode modifiers with metal oxides, carbon-based materials, MXenes, polymers, and LDH, etc., for the electrochemical detection of environmental pollutants and biomolecules and for supercapacitor applications. In addition, Ni-based bimetallic and trimetallic catalysts and their composites have been reviewed for electrochemical sensing and supercapacitor applications. The key challenges, limitations, and future perspectives of Ni-MOF-based materials are discussed. We believe that the present review article may be beneficial for the scientific community working on the development of Ni-MOF-based materials for electrochemical sensing and supercapacitor applications.

## 1. Introduction

Recently, metal–organic frameworks (MOFs) have received enormous amounts of attention because of their tunable pore structure, high surface area, and excellent thermal stability, which makes them suitable for a variety of applications such as sensing [1], catalysis [2], drug delivery [3], gas storage [4], and energy storage [5]. MOFs are a well-known emerging class of crystalline porous materials that comprise metal ions/clusters coordinated with organic ligands [6,7,8]. In particular, the nickel-based MOF (Ni-MOF) has attracted researchers’ attention owing to its unique properties such as redox activity, decent structural stability, abundant active sites, and high surface area [9,10,11]. The Ni-MOF has various advantages compared to other MOFs, including structural stability, high electrical conductivity, and redox activity of Ni centers. Therefore, due to the excellent physicochemical properties, the Ni-MOF has been widely used explored in various electrochemical applications [12,13,14]. 

In previous reports, it was revealed that environmental pollutants are major threats for the future of the world [15]. There are various environmental pollutants such as heavy metal ions (copper, lead, mercury, cadmium, etc.) [16], hydrazine [17], hydrogen peroxide [18], nitrite [19], carbendazim [20], etc., which have a negative influence on the environment and human life. Therefore, the detection of such pollutants is of great significance. Although conventional methods can be used for the determination of environmental pollutants, they have their own limitations [20,21,22]. Therefore, electrochemical technology has been developed and used for the determination of environmental pollutants [23,24,25]. Electrochemical sensing technology has several advantages such as high sensitivity, selectivity, and stability [26]. The electrochemical approach is also efficient for the determination of small biomolecules such as dopamine [27], glucose [28], uric acid [29], etc., in healthcare systems. Thus, it is of particular interest to use electrochemical sensing technology for the monitoring of environmental pollutants and biomolecules. 

Due to the rapid growth in energy consumption, energy storage devices such as supercapacitors have gained substantial interest [30,31,32]. Supercapacitors are devices that bridg the gap between conventional capacitors and batteries [33,34]. In previous studies, it was found that high-surface-area materials with a highly porous nature are beneficial for supercapacitor applications [35,36,37]. Although the Ni-MOF has a decent porous nature and high surface area, it has limitations such as low conductivity [38]. Thus, the conductivity of the Ni-MOF was improved by employing novel strategies such as doping [39] and integrating conductive materials [40].

Herein, we report the progress in the design and fabrication of Ni-MOF-based hybrid materials for electrochemical sensing and supercapacitor applications. We believe that the present review article will be useful for researchers working in the fabrication of Ni-MOF-based materials for biosensors and energy storage systems.

## 2. Ni-MOF in Electrochemical Sensing Application

Previous years have witnessed a rapid surge in the utilization of Ni-MOF-based materials for the construction of electrochemical sensors. Various hybrid composites were fabricated and employed for the determination of environmental pollutants and small biomolecules. In this section, we compile Ni-MOF-based materials for the detection of numerous analytes. 

### 2.1. Ni-MOF and Ni-MOF/Metal Oxide for Electrochemical Sensors

**Scheme 1:**
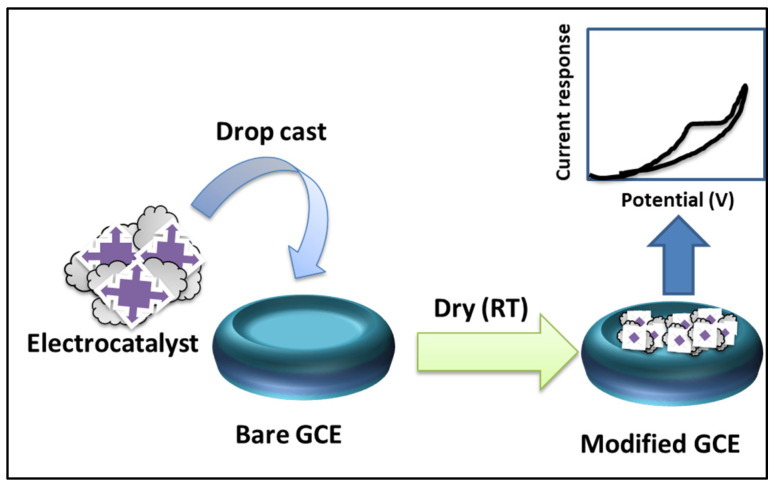
Schematic of the fabrication of electrochemical sensor.

Electrochemical sensors involve the assembly of three electrodes: the working electrodes consist of a glassy carbon electrode (GCE), a gold (Au) and indium tin oxide (ITO) screen-printed carbon electrode (SPCE), a fluorine-doped tin oxide (FTO) carbon paste electrode (CPE), whereas a platinum electrode is used as a counter electrode with silver/silver chloride as the reference electrode. A schematic for the fabrication of the electrochemical sensor is illustrated in Scheme 1. The electrochemical performance of the sensors can be evaluated in terms of the limit of detection (LOD) and sensitivity, as described in the equations given below:

LOD = 3 × σ/S(1)

Sensitivity = S/area of the electrode(2)

where σ is the standard error, and S is the slope of the calibration curve.

**Figure 1:**
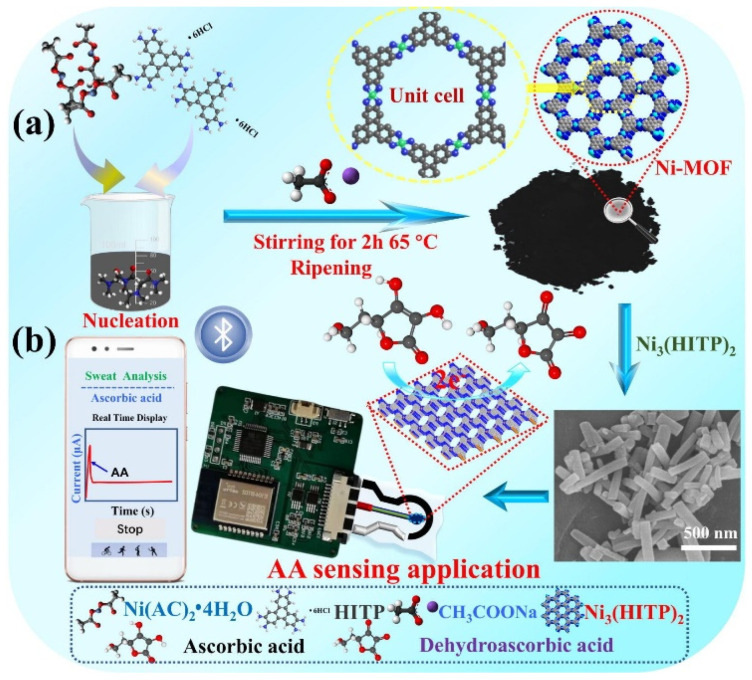
Schematic shows the structure of the Ni_3_(HITP)_2_ (**a**) and its application in sensing (**b**). Reprinted with permission from Ref. [41]. 2022, Elsevier.

Previously, Ni-MOF-based materials have been extensively used in various electrochemical sensing applications. In this connection, Wang et al. [41] reported the preparation of a Ni-MOF (Ni_3_(2, 3, 6, 7, 10, 11-hexaiminotriphenylene)_2_ (Ni_3_(HITP)_2_)). Figure 1a shows the structure of Ni_3_(HITP)_2_ and its role in the fabrication of the sensor (Figure 1b).

The prepared Ni-MOF was characterized using scanning electron microscopy (SEM) and X-ray diffraction (XRD) methods to observe the morphological and crystalline nature of the prepared Ni-MOF. XRD analysis revealed that the Ni-MOF has a decent crystalline nature, whereas SEM investigations suggested the presence of nanorod (NR)-like surface morphology. This prepared Ni-MOF was used as sensing material for ascorbic acid (AA) using the electrochemical method. The Ni-MOF-modified screen-printed carbon electrode (SPCE) has higher catalytic activity and acceptable conductivity, which improved electron transportation for the electro-oxidation of AA. The unique structure of the Ni-MOF material allowed the AA to significantly bind with active sites and enhance the detection of AA. This proposed AA sensor delivered a sensitivity of 0.814 μA μM^−1^ cm^−2^ and limit of detection (LOD) of 1 μM with a wide linear range (WDR) of 2 to 200 μM. Kavya et al. [42] proposed that a palladium (Pd)-doped Ni-MOF may show improved catalytic activities and conductivity for the electrochemical sensing of dopamine (DA) using cyclic voltammetry and differential pulse voltammetry (CV and DPV) methods. The prepared Pd@Ni-MOF-based electrode delivered a LOD of 0.01 µM and WDR of 0.001 µM to 100 µM. The presence of conductivity and synergistic interactions improved the detection of DA in urine samples and enhanced anti-interfering properties. Huang et al. [43] stated that L-tryptophan (L-TRP) can be detected using a cobalt (Co)-doped Ni-MOF (Co-Ni-MOF-1%)-modified glassy carbon electrode (GCE). It was believed that synergy effects between Ni^2+^ and Co^2+^ ions improved the electrochemical properties and surface area of the Co-Ni-MOF-1%. Therefore, Co-Ni-MOF-1–modified GCE showed a LOD of 8.7 nM and high selectivity for the sensing of L-TRP. Differential pulse anodic stripping voltammetry (DPASV) may be a sensitive technique for the electrochemical detection of heavy metal ions (HMI) and environmental pollutants. In this regard, ferrocene (Fc)-functionalized NH_2_-Ni-MOF (Fc-NH_2_-Ni-MOF) was fabricated for the monitoring of copper, lead, and cadmium (Cu^2+^, Pb^2+^ and Cd^2+^) HMI [44]. The presence of nanoplates of NH_2_-Ni-MOF may promote the adsorption of HMI, whereas Fc moiety enhances the conductivity of the Fc-NH_2_-Ni-MOF. Therefore, the Fc-NH_2_-Ni-MOF modified electrode showed a LOD of 6.3, 0.2, and 7.1 nM for the detection of Cu^2+^, Pb^2+^, and Cd^2+^, respectively. Due to the high surface area and porosity of Ni-MOF, various electrochemical sensors have been developed. With this in mind, a Ni-benzimidazole MOF has also been used for the development of a glucose sensor [45]. The money-chain-like Ni-MOF superstructure may improve the electrochemical detection of glucose, and a high sensitivity of 2199.88 mA M^−1^ cm^−2^ was obtained. A doping strategy may improve the conductivity of MOF-based materials. A silver (Ag)-doped Ni-MOF was obtained through hydrothermal and chemical reduction approaches [46]. It was observed that Ag NPs, 20 nm in size, were dispersed between the Ni-MOF nanosheets (NSs). The Ag@Ni-MOF-based electrode can monitor glucose with a LOD of 5 μM and sensitivity of 160.08 μA mM^−1^ cm^−2^. Ni et al. [47] introduced Fc derivatives into two Ni-MOFs and examined their potential for glucose monitoring. The proposed strategies showed the introduction of bidentate 1,1′-ferrocenedicarboxylic acid (1,1′-Fc) into a 3D Ni-1,4-benzenedicarboxylic acid MOF (Fc-Ni-BDC) through the benign hydrothermal method. On the other hand, ferrocenemethylamine (Fc-NH_2_) is encapsulated in the cavity of a 2D Ni-HHTP MOF (HHTP = 2, 3, 6, 7, 10, 11-hexahydroxytriphenylene) using electrostatic attraction to form Fc-Ni-HHTP. The presence of the synergy between the Fc derivatives and MOFs enhances the electrochemical properties of the proposed MOFs towards glucose detection. The Fc-Ni-BDC-based electrode can be used to determine glucose levels in human serum and coke samples with acceptable recovery. Zhang et al [48]. prepared a Ni^2+^-terephthalic acid MOF for the detection of carbamazepine (CBZ). The authors employed the square-wave voltammetry (SWV) method for the determination of CBZ using a Ni-MOF catalyst. The proposed novel Ni-MOF-based modified GCE demonstrated a LOD of 1.03 µM and LR of 20 to 300 µM with acceptable recovery in serum samples. The biological ligand (asparagine)-modified Ni-MOF (Ni-Bio-MOF) was prepared using the hydrothermal method [49]. The asparagine may create defects on the Ni-MOF surface and enhance the electrochemical capability of Ni-Bio-MOF for electrochemical sensing applications. Furthermore, a Diclofenac (DCF) polymer imprinted with L-methionine (PL-Met) was also electrodeposited on the carbon paste electrode (CPE) for the determination of DCF. The excellent LOD of 0.17 pM and sensitivity of 2015.5 μA μM^−1^ cm^−2^ were achieved for the detection of DCF. The incorporation of Ni-MOF with metal oxides may exhibit more active sites, improved conductivity, and catalytic properties. In this regard, Atefi et al. [50] prepared cerium dioxide (CeO_2_) NP-modified Ni-MOF composite through co-precipitation, hydrothermal, and electrodeposition-assisted approaches. The Ni-MOF/CeO_2_-based electrode detection limit was 0.03 mM, with high sensitivity of 2488 µA mM^−1^ cm^−2^. This novel electrochemical sensor also recovered glucose from human sweat samples. This electrode has many advantages such as high sensitivity, selectivity, stability, and fast response time. As discussed above, Ni-MOF and Ni-MOF/metal oxides exhibit various advantages for the construction of electrochemical sensors. However, some limitations remain a challenge for researchers to explore their potential for real-time sensing applications. Although pristine Ni-MOF offers excellent porosity abundant active sites for electrochemical reactions, low intrinsic conductivity is the major challenge that may limit the electron transfer rates and affects the sensitivity of the electrochemical sensors. The metal doping strategy has been explored to enhance the conductivity of the Ni-MOF, but reproducibility may be another significant challenge. In contrast, the integration of metal oxides with Ni-MOF exhibits synergistic interactions with better charge transfer and more active sites. Thus, improved selectivity and stability have been observed for Ni-MOF/metal oxide-based sensors compared to pristine Ni-MOF-based sensors.

### 2.2. Ni-MOF/GO, rGO, CNT, and gCN Materials for Electrochemical Sensors 

**Figure 2:**
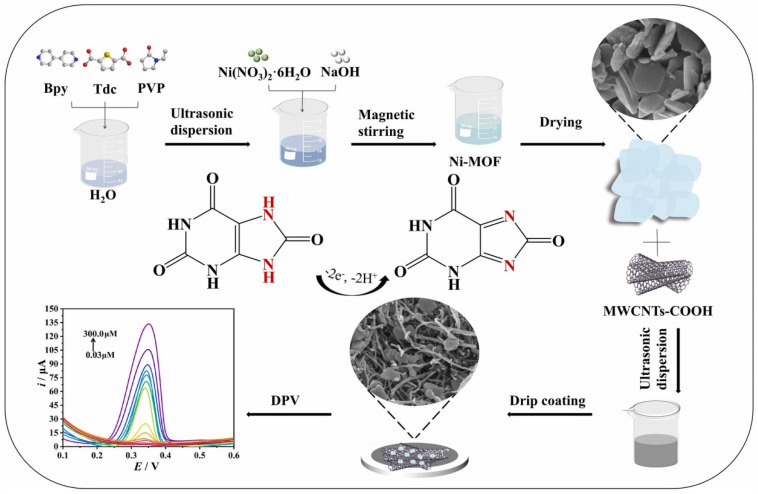
Pictorial representation of the fabrication of Ni-MOF/MWCNTs-COOH/GCE towards UA detection. Reprinted with permission from Ref. [54]. 2024, Elsevier.

In a recent study [51], Ni-MOF was prepared using the hydrothermal method and modified with reduced graphene oxide (rGO). The presence of rGO may enhance the electrical conductivity of the Ni-MOF/rGO composite. In addition, the presence of synergistic interactions such as high surface area and improved electrochemically active sites facilitated the redox reactions for the determination of epinephrine (EP) and folic acid (FA). This Ni-MOF/rGO-modified GCE delivered a LOD of 0.018 and 0.016 µM for EP and FA detection, respectively. Human serum- and urine sample-based studies revealed the recovery of acceptable amounts of EP and FA, indicating its potential for large-scale applications. The incorporation of Ni-MOF with multi-walled carbon nanotubes (MWCNTs) may be promising strategy. In this regard, Ni-MOF/MWCNT was explored as a sensing layer for the detection of glutamate using the amperometry (Amp.) method [52]. This proposed glutamate sensor was capable of determining glutamate with a LOD of 69.9 μM and sensitivity of 2.90 × 10^2^ μA mM^−1^ cm^−2^. 4-Chlorphenol (4-CP) is one of the environmental pollutants that needs significant attention. The detection of 4-CP is of particular significance. Therefore, a Ni-BDC (BDC: 1, 4-dicarboxybenzene)-MOF was incorporated with MWCNTs for the determination of 4-CP [53]. A detection limit of 16.5 nM and LR of 50 to 500 µM with strong stability and excellent selectivity were obtained for 4-CP detection. A uric acid (UA) sensor was also fabricated by employing Ni-MOF/MWCNTs-COOH-modified GCE [54]. The preparation of Ni-MOF/MWCNTs-COOH and its application in UA sensing is illustrated in Figure 2. The MWCNTs-COOH reduced the accumulation of Ni-MOF, which improved the active sites and surface area of the Ni-MOF/MWCNTs-COOH. The Ni-MOF/MWCNTs-COOH modified electrode-based UA sensing exhibited a LOD of 0.005 μM with sensitivity of 6.377 µA µM^−1^.

Tan et al. [55] fabricated a kaempferol (KA) electrochemical sensor by employing β-cyclodextrin/fullerene-graphene oxide/Ni-MOF (β-CD/C_60_-GO/Ni-MOF) as electrode material. This β-CD/C_60_-GO/Ni-MOF-based electrode exhibits improved conductivity, active sites, and high surface area, which realize the detection of KA with a LOD of 58 nM. A highly dense Ni-MOF nanoflake array-supported graphene/carbon fiber (Ni-MOF/rGO/CF) composite-based flexible microelectrode was explored for the electrochemical detection of glucose [56]. This novel glucose sensor demonstrates a LOD of 0.6 µM and sensitivity of 852 µA cm^−2^ mM^−1^ with a wide LR of 6 µM to 2.09 mM. In another previous study [57], Ni-MOF intercalated with amino acid-functionalized graphene nanoplatelets (FxGnP-Ni-MOF) was prepared using a hydrothermal-assisted synthetic approach. It was found that hydroxyl/epoxy functional groups in FxGnP may acts as nucleation centers that restrict the uncontrolled growth of Ni-MOF. Therefore, the FxGnP-Ni-MOF modified electrode exhibited enhanced electrochemical properties for the detection of bisphenol A (BPA). An interesting LOD of 0.184 nM and high sensitivity of 47.65 μA mM^−1^ cm^−2^ were obtained for BPA sensing. On the other hand, Mxenes are layered two-dimensional materials that possess excellent conductivity and electronic properties, which makes them a promising conductive support for the preparation of hybrid composite materials. Yan et al. [58] reported the fabrication of a novel titanium carbide modified Ni-MOF composite (Ti_3_C_2_@Ni-MOF). The constructed Ti_3_C_2_@Ni-MOF-2@CC electrode was adopted as an electrochemical sensor for the simultaneous detection of adenine (A) and AA. The integration of MXene to the Ni-MOF enhances the electrochemical sensing properties of the optimized Ti_3_C_2_@Ni-MOF-2@CC electrode. This Ti_3_C_2_@Ni-MOF-2@CC electrode also demonstrated excellent anti-interference properties and reproducibility. It can be understood that Ni-MOF/rGO composites exhibit enhanced electrical conductivity and a larger surface area. Thus, these are Ni-MOF/rGO composite-based electrodes. Thus, improved electrochemical performance was observed for the determination of analytes such as epinephrine and folic acid. However, the synthesis of rGO involves harsh conditions, which in not environmentally friendly. The Ni-MOF/MWCNT hybrid materials show that the presence of MWCNTs reduces the aggregation of Ni-MOF and improves the electron transfer for the determination of target analytes such as UA and 4-CP. Unfortunately, the functionalization of CNTs is complex and its dispersibility remains a key challenge. On the other hand, other hybrid composites such as β-CD/C60-GO/Ni-MOF and Ni-MOF/rGO/CF also showed promising electrochemical activity, but structural complexity and scalability are the significant challenges that need to be overcome.

### 2.3. Bimetallic Ni-Based MOFs for Electrochemical Sensing Application

**Figure 3:**
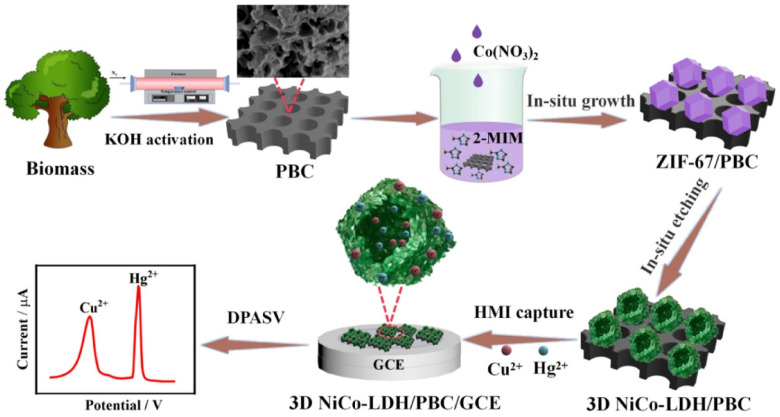
Schematic of the fabrication of 3D NiCo-LDH/PBC/GCE for Cu^2+^ and Hg^2+^ detection. Reprinted with permission from Ref. [64]. 2024, Elsevier.

Carbendazim (CBM) is a fungicide used in the cultivation of vegetables and fruits. However, it may cause various negative effects on human health such as infertility in the human body and thyroid and liver damage. In this regard, a novel Ni- and iron (Fe)-based bimetallic MOF structure was developed for the construction of a CBM sensor [59]. The solvothermal method was employed for the preparation of a pebble-like bimetallic Ni-Fe(PDC) MOF. This bimetallic MOF-anchored graphite electrode can improve the electron transfer and kinetics of the fabricated electrode towards the detection of CBM. DPV-based studies revealed that the fabricated CBM sensor exhibits a LOD of 2.3 nM and limit of quantification (LOQ) of 7.7 nM via DPV. As mentioned above, this bimetallic Ni-based MOF has been explored for electrochemical sensing applications, exhibiting remarkable sensitivity and selectivity for the targeted analytes. The Ni-Fe(PDC) MOF offers decent electron transfer and reasonable LOD for the detection of CBM, but the synthesis of this bimetallic MOF involves 24 h, which needs to be reduced for scalability [59]. In another previous work [60], a flexible electrochemical sensor was developed using novel bimetallic NiCo-MOF-derived NiCoSe as the electrode modifier. The preparation of NiCoSe involved two-step synthesis approaches and is characterized by XRD, which confirmed the formation of NiCoSe with phase purity. NiCoSe/CC was used as an electrochemical sensor for acetaminophen (AC) detection, and an interesting LOD of 0.012 μM and high sensitivity of 0.147 μA μM^−1^ were achieved. Due to the excellent performance of the above electrodes fabricated for AC detection in tablet and environmental samples, it is worth mentioning that MOF-derived materials may be explored for practical applications. Numerous efforts have been significantly employed to develop highly selective and sensitive MOF-based electrochemical sensors. The NiCo-MOF/MWCNT- and benzoyl Fc (BFC)-based sensor was developed for the detection of _L_-cysteine (_L_-Cys) and TRP [61]. It was found that NiCo-MOF/MWCNTs/BFC/CPE displayed decent repeatability, appropriate selectivity, and acceptable reproducibility for the detection of TRP and _L_-Cys. The LOD of 0.015 and wide LR of 0.05 to 1000 µM were achieved for _L_-Cys detection. The improved catalytic behavior of the fabricated electrode also detected TRP and _L_-Cys in human urine and blood serum samples with satisfactory results. Despite the multi-analyte detection properties of the NiCo-MOF/MWCNTs/BFC composite, the proposed sensor has limitations for practical applications such as selectivity in real samples. It is understood that excessive intake of fructose may cause various diseases such as metabolic disorders and obesity. It is really of great interest to fabricate a fructose sensor using efficient sensing materials. CoNi-MOF was synthesized in situ on nickel foam (NF) using the hydrothermal method [62]. The synergy interactions between the self-supported NF and CoNi-MOF may enhance sensitivity of CoNi-MOF towards fructose. Therefore, the proposed fructose sensor displayed a detection limit of 2.53 µM with high sensitivity and reproducibility. Another work [63] also reported the preparation of Ni/Zn-MOF through the one-step solvothermal method. Furthermore, Ni NPs were incorporated using the pyrolysis approach in nitrogen atmosphere. Subsequently, Ni_3_ZnC_0.7_/Ni was obtained, and its application for the detection of HQ and CC was explored using the DPV technique. LODs of 0.14 µM and 0.21 µM were obtained for HQ and CC detection with acceptable recovery of HQ and CC in yellow river and tap water samples. The Ni/Zn-MOF-derived Ni_3_ZnC_0.7_/Ni enables the simultaneous detection of HQ/CC, but pyrolysis steps introduce energy costs. A hollow three-dimensional (3D) NiCo-LDH nanocages/porous biochar (PBC) composite was also prepared under facile conditions for the detection of Cu^2+^ and Hg^2+^, as illustrated in Figure 3 [64]. The 3D NiCo-LDH/PBC-based electrode could deliver an interesting LOD of 0.03 µg/L and 0.03 µg/L for the detection of Cu^2+^ and Hg^2+^, respectively. The 3D NiCo-LDH/PBC composites exhibited ultra-low LODs for the detection of Cu^2+^ and Hg^2+^ ions, but biochar quality control is critical for practical applications. In another study, CuNi-MOF was combined with rGO, which was also explored as a promising electrode material for the determination of UA and DA [65]. The wearable biosensor exhibited sensitivity of 0.019 μA μM^−1^ cm^−2^ and 0.026 μA μM^−1^ cm^−2^ for the determination of DA and UA, respectively. Li et al. [66] developed hepatitis B surface antigen (HBsAg) using a gold (Au)/cobalt oxide (Co_3_O_4_) NP-functionalized NiCo (HITP)-MOF as electrode material, which exhibited a LOD of 15 fg/mL. The conductive nature and improved catalytic properties of the fabricated electrode material enhanced the selectivity and sensitivity for HBsAg detection. The acceptable recovery of real samples also suggests its potential for practical applications.

**Figure 4:**
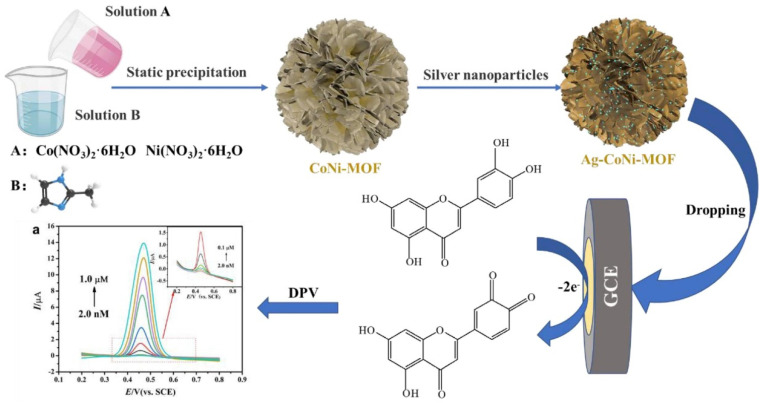
Schematic representation of the fabrication of the Ag-doped CoNi-MOF-based luteolin sensor. Reprinted with permission from Ref. [77]. 2022, Elsevier.

The MOF-derived materials exhibit a large surface area and improved porosity, which makes them efficient materials for sensing applications. Previously, Ni-MOF (2, 3, 6, 7, 14, 15-hexahydroxy triptycene (Ni-HHTT)) was used as a precursor to obtain the nickel phosphide (NiP) on NF [67]. The obtained NiP/NF was used for glucose detection, which displayed a LOD of 0.15 µM and wide LR of 0.5 to 1700 and 1700 to 4100 µM. In a previous report [68], chloramphenicol (CAP) was also developed by employing a NiCo(BTC = 1,3,5-benzenetricarboxylicacid)-MOF/rGO composite. The prepared electrode shows improved conductivity, large surface area, and abundant active sites for electrochemical reactions. Therefore, the fabricated electrode could determine CAP in real samples. The LOD of 0.235 μM and high sensitivity of 33.12 μA μM^−1^ cm^−2^ with a wide LR of 0.1 to 100 μM were also observed under the facile conditions. Gao et al. [69] used rGO- and CoNi-MOF-based composite material for the detection of hippuric acid (HA). The rGO/CoNi-MOF based moleculary imprinted electrochemical (MIP) sensor was developed, which was able to monitor the HA in human urine sample with satisfactory recovery. As mentioned in the above reports, bimetallic MOFs have improved catalytic properties and sensing behavior for the construction of various electrochemical sensors. Thus, CoNi-MOF was also synthesized using the hydrothermal method [70]. The CoNi-MOF was combined with chitosan-polyacrylamide (CS-PAM) and adopted as sensing material for adrenaline detection, which displayed decent selectivity, stability, a wide LR of 0.5 to 10 μM and 10 to 2500 μM, and a LOD of 0.167 μM. Sun et al. [71] also proposed the fabrication of alpha-fetoprotein (AFP) electrochemical sensor utilizing D flower-like CoNi-MOF and sea urchin-like PdCuNi materials, which delivered acceptable electrochemical performance in terms of sensitivity and selectivity. In another research study [72], it was observed that a hydrothermally synthesized Fe-rich FeCoNi-MOF on NF possess excellent crysallinity, more active sites, and a tighter structure. The fabricated electrode was employed as a working electrode for the detection of imidacloprid in fruits, displaying a decent LOD of 0.04 pM and wide LR of 1 pM to 120 µM. Real sample-based studies in fresh tea, apple, cucumber, and tomato suggested its potential for commercialization. In another report, zirconium dioxide (ZrO_2_)@NiCo-MOFs@Au NPs were employed for the detection of Cd^2+^ and *S. aureus* [73]. The high surface area and amplification technology enhance the electrochemical detection of Cd^2+^ and *S. aureus*. The proposed sensor was also efficient for the determination of Cd^2+^ and *S. aureus* in fish and scallops. NiCo-MOF/CNTs/graphene nanosheet-printed polyethylene terephthalate (GNP) was also employed as a peroxidase mimic sensor for the determination of alpha-fetoprotein (AFP) [74]. Wang et al. [75] found that FeNi-MOF-derived materials exhibit high sensitivity for hydrazine detection, whereas kanamycin (Kan) sensor was developed using a CoNi-Bio-MOF and Au-doped coral-like ZrO_2_-based electrode [76]. Ag-doped 3D CoNi-MOF was explored as sensing material for the determination of luteolin, as displayed in Figure 4 [77]. It was observed that the incorporation of Ag into a 3D flower-like hierarchical structure enhanced the specific surface area and electrical conductivity. This may further increase the active sites for electrochemical reactions and enhance the sensitivity of the modified electrode for the detection of luteolin.

A norfloxacin (NFC) sensor was also fabricated by employing a hydrothermally prepared NiCo-MOF/NF electrode [78]. The NiCo-MOF was derived from NiCo double-layered hydroxide (LDH) through simple strategies. This sensor exhibits excellent detection of NFC in pharmaceutical blood serum and urine samples. Pan et al. [79] prepared a series of 2D/3D hierarchical NiCu-MOF-_x_ (where x = 2, 4, 6, and 8) using the hydrothermal method. The optimized NiCU-MOF-6-modified GCE demonstrated a wide LR and high sensitivity for glucose detection. This enhanced detection of glucose may be attributed to the presence of a larger specific surface area, interconnected channels for electron transfer, and electrochemically active sites. Satisfactory results for real-sample studies were also observed with human serum samples. In another work [80], CoNi-MOF (Co_x_Ni_3-x_(HITP)_2_) was synthesized using an organic ligand (2, 3, 6, 7, 10, 11-hexaaminotriphenylene (HATP)). It can be seen that the abovementioned ligand has mixed metal valances (Co^2+^/Co^3+^ and Ni^2+^/Ni^3+^) with more defects in the skeleton of the NOF structure. This fabricated MOF was employed as sensing material for enrofloxacin detection, which exhibited a LOD of 0.2 fg/mL and high selectivity. This reveals that the ligand plays a crucial role and affects the physicochemical properties of the MOF structure. The direct growth of the NiCo-(HHTP)MOF on CC through the hydrothermal method [81]. The synergistic effects of Ni and Co elements and NiCo-(HHTP)MOF provide a high surface area and enhanced active sites, which boost the charge transport and electrochemical sensing properties. The optimized results exhibited a LOD of 100 nM and LR of 0.3 μM to 2.312 mM with sensitivity of 3250 μA mM^−1^ cm^−2^ for the detection of glucose. It is clear that bimetallic Ni-MOFs display enhanced electrochemical activities with structural complexity but face challenges in scalability, eco-friendly synthesis, and long-term stability in real-world sensing environments. Future research may focus on these key points for the fabrication of bimetallic MOF-based electrochemical sensors. 

### 2.4. Other Ni-MOF-Based Materials 

**Figure 5:**
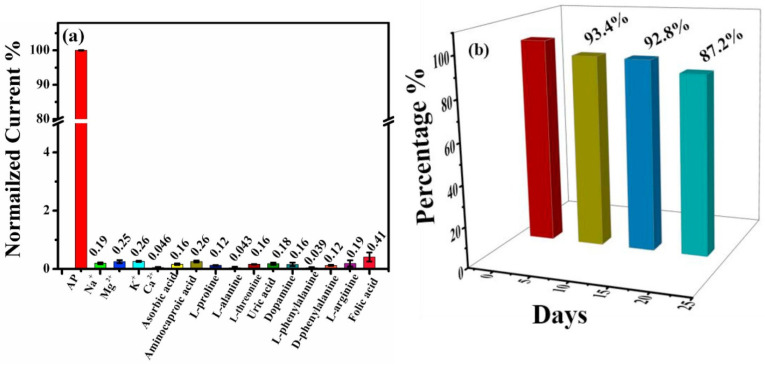
(**a**) Selectivity and (**b**) long-term stability of AP sensor. Reprinted with permission from Ref. [96]. 2021, Elsevier.

Li et al. [82] proposed a novel nonlinear and non-enzymatic glucose sensor and explored the Langmuir relationship for glucose-sensing applications. The Ni^3+^-Ni-MOF@Ni_3_(PO_4_)_2_ was prepared and adopted as glucose-sensing material. The authors reported a wide LR of 1 to 9000 µM by employing the Langmuir relationship for glucose detection. A novel BPA sensor was also developed by exploring 2 D MOF-derived Ni_2_P@C material [83]. The proposed BPA sensor exhibited a LOD of 56.8 nM, sensitivity of 0.951 μA cm^−2^ μM^−1^, and LR of 1 to 100 μM. The high selectivity for BPA detection may be attributed to the presence of improved surface area, active sites, and synergy of the prepared Ni_2_P@C material. In another study [84], a nickel–niacin MOF (NiNiacin-MOF) was modified with CPE and utilized as a working electrode for glucose detection in human blood sample. The authors observed that the modified electrode has good sensitivity for glucose detection, and a decent LOD of 0.006 mM was achieved. A yolk–shell (YS)-Ni@NC_600_ was obtained using a core–shell precursor of Ni-MOF@COF_TAPB-DVA_ [85]. The YS)-Ni@NC_600_ modified electrode was explored for DA sensing, and a LOD of 0.041 µM and LR of 0.136 to 70 µM were obtained with excellent selectivity and stability. Zhu et al. [86] developed a novel MIP biosensor using Ni_3_(HITP)_2_-MOF as sensing material. The glucose sensing studies were performed using the abovementioned MOF, delivering a LOD of 0.31 μM and LR of 1 μM to 100 mM. The human blood sample-based recovery of glucose suggests its potential for practical applications. The MOF-derived graphitic carbon-decorated nickel vanadate (NiVO_4_) was fabricated for the construction of sarcosine [87]. In brief, multiple steps were used for the preparation of a Ni-BDC MOF, followed by vanadium (V) doping. The obtained V-doped MOF was calcinated to obtain the GC@NiVO_4_ composite. The MOF-derived GC@NiVO_4_ composite exhibited a LOD of 0.03 µM and decent recovery of 95 to 105% in real-sample investigations. A wearable Ni-MOF-based electrochemical sensor integrated with janus fabric for the determination of AA and glucose in sweat [88]. It is expected that the proposed sensor may be employed in practical applications such as a clinical care system. A Ni-MOF was also grown as a carbonized loofah sponge towards the detection of glucose [89]. Authors have investigated the effects of carboxylic acid ligands and temperatures on the electrochemical sensing performance of the fabricated glucose detection. The optimized conditions revealed that a LOD of 0.4 µM and LR of 4 µM to 2.238 mM were achieved for glucose detection. The MIP-based honeycomb-like Ni-MOF decorated with Ag NPs, and a N-doped graphene quantum dot (GQDs) composite was adopted as sensing material for olaquindox (OLA) detection in animal–origin food samples [90]. In another study [91], Ni-MOF/Fe-MOF-5 hybrid Au NPs were combined with polythionine (pTHi) for the sensitive detection of chlorpromazine (CPZ). The optimized electrode (MIP/pTHi/Ni-MOF/Fe-MOF-5/AuNPs/GCE) was highly sensitive and selective for the determination of CPZ with an excellent LOD of 0.025 µM. A novel β-cyclodextrin/Ni-MOF/GCE was also adopted as DA sensing material [92]. The Ni-MOF was prepared using the hydrothermal method, which was subsequently combined with β-cyclodextrin. This kind of electrode material exhibits enhanced conductivity and more active sites for electrochemical reactions. Therefore, it is suggested that such electrodes may be used for the determination of small molecules such as DA. Therefore, an optimized electrode shows a LOD of 0.227 µM and LR of 0.7 to 310.2 µM for DA detection using the DPV method. Wei et al. [93] employed a bimetallic CoNi-MOF and Au NP modified electrode for the electrochemical determination of enrofloxacin (ENR). It was also found that CoNi-MOF has waxberry like surface morphology. The presence of the Au NPs improved the conductivity and catalytic properties of the CoNi-MOF. The sulfhydryl group-modified aptamer (Apt)/AuNPs/CoNi-MOF/GCE displayed a LOD of 3.3 × 10^−4^ pg/mL. In another report [94], nanorod-like nickel phosphate (r-NiPO) was also prepared using MOF-derived material. This MOF-derived material exhibits improved sensitivity of 3169 μA mM^−1^ cm^−2^ for glucose detection. Pang et al. [95] employed a novel bimetallic porphyrin TCPP(Ni)-Co MOF for the electrochemical sensing of theophylline. The TCPP(Ni)-Co MOF was synthesized by employing the reflux and hydrothermal method. The TCPP(Ni)-Co MOF-modified GCE exhibits high sensitivity for the determination of theophylline, and an interesting LOD of 3.3 nM was achieved. It was believed that the coordination of Ni^2+^ ions in high electron affinity may generate a more electron-deficient environment in a conjugate TCCP ligand. This may reduce the electron compensation to Co^2+^ for the formation of Co-COO coordination bond. Therefore, it enhances the sensing performance of TCPP(Ni)-Co MOF-modified GCE for theophylline detection. In another previous work [96], Ni-doped nanoporous carbon (Ni/C) was obtained through one-step calcination of Ni-MOF. Authors have observed that the obtained Ni/C-400 material modified GCE was utilized as an electrochemical sensor for the determination of AP. The proposed sensor demonstrated excellent selectivity (Figure 5a) and long-term stability (Figure 5b) for the determination of AP. The applied AP sensor also displayed satisfactory results in human serum and urine samples without any interference. This reveals its potential for practical applications for the measurement of therapeutic drugs. 

Zhang et al. [97] also explored Ni-MOF-derived NiO nanostructured materials for the electrochemical detection of glucose. The MOF-derived nanoporous NiO NRs exhibited decent reproducibility and a wide LR, which suggests that it can be used for glucose detection in practical applications in the future. Li et al. [98] employed Ni_2_P/C as electrode material for the monitoring of UA. The Ni_2_P/C was obtained through the calcination of Ni-MOF-74 with adsorptive red phosphorus. In another previous research, Ni-MOF was in situ grown on MoS_2_ sheets to obtain the MoS_2_@Ni-MOF [99]. This synthesized MoS_2_@Ni-MOF was used as a precursor for the preparation of MoS_2_/Ni(OH)_2_. The MoS_2_/Ni(OH)_2_ modified electrode could detect the HQ and CC simultaneously. LODs of 0.43 µM and 0.48 µM were observed for HQ and CC detection, respectively. It is worthy mentioning that Ni^3+^–Ni-MOF@Ni_3_(PO_4_)_2_ offers a wide linear range for glucose detection using Langmuir relationship modeling, but its fabrication procedure may be complex with low scalability for the real-time sensing of glucose. The MOF-derived Ni_2_P@C also required a high-temperature calcination process, which limited its application for large-scale applications. The yolk–shell Ni@NC600 involves multi-step synthesis, which may hinder cost-effectiveness for mass production. The GC@NiVO_4_ composites also involved multiple doping and calcination steps. The Ag NP- and GQD-decorated Ni-MOF materials demonstrate decent electrochemical properties, but control of the uniformity of such composites is critical. The CoNi-MOF/AuNP aptasensors delivered excellent sensitivity for the determination of antibiotics due to their waxberry-like morphologies. However, storage stability and aptamer cost may limit their potential in real-time sensing application. Despite limitations, Ni-MOF-based materials are promising electrode materials for electrochemical sensing applications. The performances of the various reported Ni-MOF-based electrochemical sensors are presented in Table 1.

## 3. Ni-MOF in Supercapacitor Application

### 3.1. Ni-MOF and Ni-MOF/Metal Oxides 

**Figure 6:**
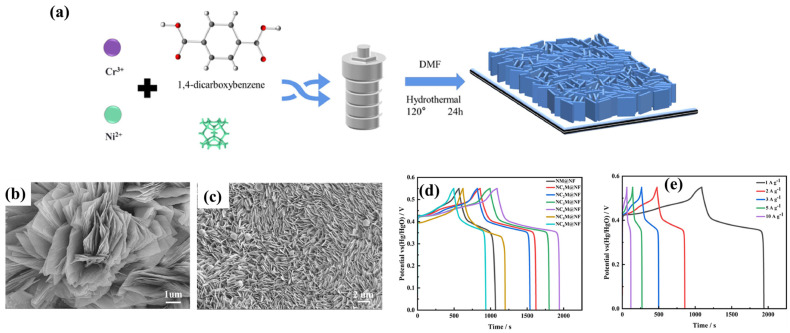
(**a**) Schematic for the synthesis of Cr^3+^-doped Ni-MOF. SEM image of (**b**) NM@NF and (**c**) NC_4_M@NF. (**d**) GCD curves of various fabricated electrodes (NM@NF, NC_1_M@NF, NC_2_M@NF, NC_3_M@NF, NC_4_M@NF, NC_5_M@NF, and NC_6_M@NF) at 1 A/g. (**e**) GCD curves of NC_4_M@NF at different current densities. Reprinted with permission from Ref. [103]. 2023, Elsevier.

In recent years, it has been observed that Ni-MOF-based materials are desirable electrode modifiers for supercapacitors and energy storage applications. With this in mind, Xia et al. [100] used a 2D unsaturated Ni-MOF for supercapacitors. The Ni-MOF was obtained using the solvothermal method, and its electrochemical properties were evaluated in a three-electrode system. The specific capacity of 746 C/g was obtained at a current density of 1 A/g. The improved performance of the Ni-MOF-based supercapacitors was attributed to the presence of 2D hierarchical nanosheets, which may provide abundant electrochemically active sites and improved electrical conductivity. In another study [101], the solvothermal method was explored for the preparation of Ni-MOF on stainless steel substrate, and a specific capacitance of 850.42 F/g was obtained at 1 mA/cm^2^. This indicates that Ni-MOF is promising electrode material for supercapacitors. The inherent conductivity and stability of the electrode materials are promising characteristics for supercapacitor application. The conductivity of the Ni-MOF can be improved with the doping strategy; therefore, a lanthanum (La)-doped Ni-MOF was fabricated through the one-pot hydrothermal method [102]. The 10 wt% La-doped Ni-MOF was found to be an optimized material that exhibited a specific capacitance of 159.9 mA H/g at 1 A/g. In another report [103], it was observed that hydrothermally prepared Ni-MOF consists of a nanoflower-like structure with 3D nanosheet stacking on the NF electrode (Figure 6a). Chromium (Cr^3+^) was added to further improve the electrochemical activity of the Cr^3+^-doped Ni-MOF (NC_4_M@NF). A different percentage of Cr^3+^ was used for the preparation of Ni-MOF samples, which can be labeled as NM@NF, NC_1_M@NF, NC_2_M@NF, NC_3_M@NF, NC_4_M@NF, NC_5_M@NF, and NC_6_M@NF. SEM images of the NM@NF and NC_4_M@NF are displayed in Figure 6b and 6c, respectively. The NM@NF material was composed of 3D nanosheets, which are uniformly grown on the surface of the NF electrode. However, the surface of the nanosheets was found to be uneven. The nanosheets were stacked to form a thick lamellar array that finally formed a nanoflower-like structure. In the case of the NC_4_M@NF, it was observed that the growth of the NC_4_M@NF nanosheets was not random. It was perpendicular to the NF skeleton. This reveals that the incorporation of Cr^3+^ generated a nanosheet-like structure with favorable orientation to the substrate. The galvanostatic charge–discharge (GCD) curves of the various fabricated electrodes at 1 A/g are shown in Figure 6d. It is observed that NC_4_M@NF has higher electrochemical performance compared to the other electrodes. The GCD curves of the NC_4_M@NF at different current densities are displayed in Figure 6e. It was found that NC_4_M@NF has high specific capacity at 1 A/g. NC_4_M@NF-based electrochemical investigations have suggested that a specific capacity of 853 C/g can be obtained at 1 A/g. In contrast, an asymmetric NC_4_M@NF//AC device was also fabricated, which delivered a specific capacitance of 160.47 F/g at 1 A/g.

The microwave method was also employed for the preparation of Ni-MOF using nickel nitrate hexahydrate and p-benzenedicarboxylic acid [104]. It was found that different surface morphologies such as flakes, plates, nanoflowers, and globules can be obtained by tuning various parameters such as temperature and precursor concentration. It was stated that flake and plate-like Ni-MOF materials have a 2D structure, whereas nanoflower- and globule-like Ni-MOFs were found to be 3D structured materials. Authors have found that flake- and plate-like Ni-MOF exhibited decent specific capacitance, which was directly proportional to the surface area, whereas globule- and nanoflower-based studies have exhibited different patterns for specific capacitance. The surface areas of the globules and nanoflowers were almost the same, but globule-structured materials demonstrated higher specific capacitance, which may be attributed to its higher electrical conductivity. Another study also revealed that Ni-MOF can be employed for supercapacitor application due to its larger surface area and porosity [105]. Authors have achieved specific capacitance of 2567.23 F/g at 2 A/g in a hydrochloric acid system. A benzene-1, 4-dicarboxylic acid-based Ni-MOF exhibited an excellent surface area of 1456.10 m^2^/g and pore size volume of 1.25 cm^3^/g [106]. The presence of high surface area and porosity enhances the specific capacity to 678.23 C/g at 3 mV/s in a three-electrode system. Authors have also fabricated asymmetric supercapacitors that delivered specific capacity of 197.20 C/g and energy density/power density of 46.56 Wh/kg/850 W/kg. The solvothermal method-based synthesis of Ni-BDC MOF was prepared for supercapacitor application [107]. Authors have used different solvent compositions for the preparation of Ni-BDC MOF. Electrochemical studies have revealed that specific capacitance of 1124 F/g was obtained at 10 A/g with power density of 3040.7 W/Kg and energy density of 87 Wh/Kg. Different metal concentrations (1M (NM-1), 2M (NM-2), 3M (NM-3), and 4M (NM-4)) were also used for the solvothermal synthesis of Ni-MOF [108]. NM-3- and NM-4-based materials form a sheet-like structure of Ni(OH)_2_ LDH and offer more active sites and improved surface area/pore size, which facilitate the electron transport process. The high specific capacitance of 1668 F/g and specific capacity of 294 mA /g at 10 A/g for NM-3-based electrode. A vanadium oxide (V_2_O_5_) nanosheet/Ni-MOF was also fabricated using facile conditions [109]. The proposed material exhibited a specific capacitance of 546 F/g at 1 A/g under the optimized conditions. Rajamany et al. [110] mentioned that MOF suffers from the limitation of poor conductivity and cyclic stability for long-term applications. In this context, nitrogen (N)-enriched Ni-MOF was prepared through the benign solvothermal method using polyvinylpyrrolidone (PVP) as an N source and structure-directing agent. It was found that use of PVP alters the flower-like surface structure of the Ni-MOF to a hierarchical micro-spherical structure. Therefore, the N-Ni-MOF-based electrode could deliver a specific capacitance of 1519 F/g at 1 A/g with a reasonable stability of 2000 cycles. The synthetic methods also affect the properties of the electrode materials. In this regard, the ultrasonication approach was adopted for the formation of a petal-like Ni-MOF structure [111]. The Ni-MOF shows improved porosity and surface area, thereby obtaining an interesting specific capacitance of 221 F/g at 1 A/g with a stability of 2000 cycles. The inherent conductivity of the Ni-MOF can be further improved. The vanadium (V)-doped Ni-MOF with 3D hydrangea-like surface morphology was obtained through the one-pot hydrothermal synthesis method [112]. The obtained Ni_0.9_V_0.1_-MOF exhibited a specific capacitance of 1182 F/g at 1 A/g. The incorporation of V into the Ni-MOF structure enhances the conductivity and improves the electrochemical properties. The formation of hybrid composites of Ni-MOF is of great importance. The ZnCo_2_O_4_@Ni-MOF (ZCO@Ni-MOF) core–shell structure was also fabricated on the NF electrode for supercapacitor application [113]. The ZCO@Ni-MOF exhibits an interesting specific capacitance of 1800 F/g at 2 A/g in the presence of a 1 M potassium hydroxide (KOH) electrolyte system. The presence of synergism between the ZCO and Ni-MOF may be the key points for this interesting specific capacitance. The pristine Ni-MOFs, particularly the 2D and 3D nanostructured Ni-MOFs, offer a high specific surface area, more redox-active sites, and tunable morphologies, such as nanosheets, nanoflowers, flakes, etc. But the low conductivity of the pristine Ni-MOFs is the major concern, which is overcome by introducing doping with metals such as La, Cr, and V. However, Ni-MOF/metal oxide composites show better electrochemical performance for supercapacitors. The presence of synergistic interactions between the metal oxides and Ni-MOF improves the charge transfer and enhances specific capacitance. However, long-term stability for supercapacitors remains a key challenge for the scientific community.

### 3.2. Ni-MOF/Carbon-Based Materials

**Figure 7:**
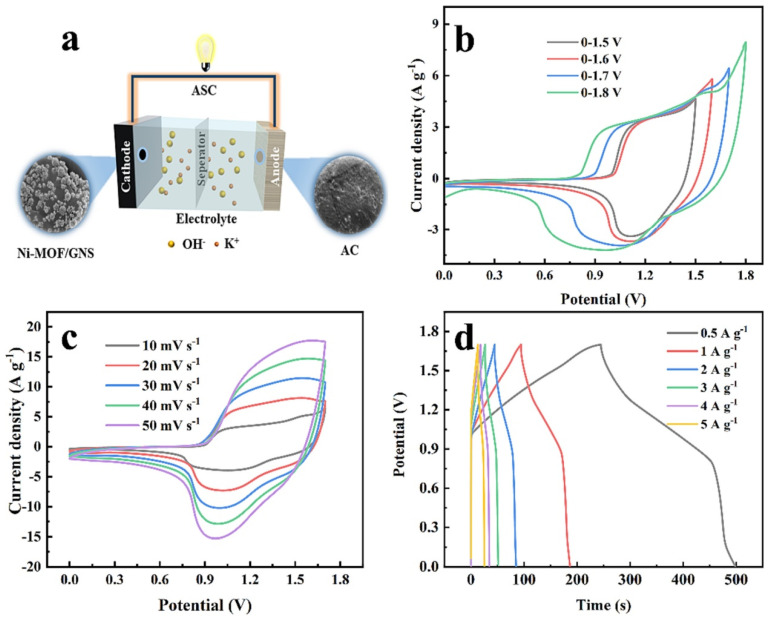
(**a**) Schematic of the S-3//AC asymmetric supercapacitors. (**b**) CV curves of S-3//AC asymmetric supercapacitor devices at 10 mV/s. (**c**) CV curves of S-3//AC asymmetric supercapacitors at different scan rates. (**d**) GCD curves of S-3//AC asymmetric supercapacitors at different current densities. Reprinted with permission from Ref. [114]. 2023, Elsevier.

Carbon derivatives such as graphene, carbon nanotubes, graphitic carbon nitride (g-CN), etc., are promising supporting materials for enhancing the conductive properties of the MOF and metal oxides. With this in mind, graphite nanosheets (GNSs) were decorated with Ni-MOF to form the Ni-MOF/GNS composite using the one-pot solvothermal method [114]. The proposed novel Ni-MOF/GNS composite-based electrode exhibits a specific capacity of 737 C/g at 1 A/g with a retention rate of 61.1% after 10,000 cycles. Authors have also fabricated a S-3//AC asymmetric supercapacitor device under facile conditions. Figure 7a shows the schematic of the structure of the proposed supercapacitors, whereas CV curves of the S-3//AC asymmetric supercapacitor device at 10 mV/s are shown in Figure 7b. The potential window was optimized by employing different potential windows. After optimization, CV curves of the S-3//AC asymmetric supercapacitors were also obtained at different scan rates, as shown in Figure 7c. The GCD curves of the S-3//AC asymmetric supercapacitors were also obtained at different current densities, as displayed in Figure 7d. It was found that the S-3//AC device exhibited a higher specific capacity at 0.5 A/g.

The LDH materials are metal hydroxides with the presence of two or more metals. The LDH materials have limited electrochemical conductivity and poor reaction kinetics. The MWCNTs can be used as conductive support to improve the conductivity of the low-conducting materials. Therefore, a novel electrode material consisting of NiCo LDH, MWCNTs, and Ni-MOF was fabricated via in situ chemical reaction [115]. The Ni-MOFs/MWCNTs@NiCo-LDH-based electrode exhibited enhanced conductivity, more active sites, and electrochemical activities for supercapacitor application. Thus, an impressive specific capacity of 1032 C/g was observed at 1 A/g under the optimized conditions. Yazdani et al. [116] reported the formation of a Ni-MOF/MWCNT composite by employing a mixed ligand strategy with the hydrothermal method. The authors partially replaced benzoic acid (BA) with benzene-1, 3, 5-tricarboxylic acid (BTC) to fabricate the Ni-MOF. It is believed that the use of BTC may form the Ni-MOF with flower-like microspheres and reduce the ion diffusion path. In addition, use of the BA with MWCNTs and Ni-MOF improves the electrical conductivity of the electrode material. The obtained material exhibits more active sites, improved stability and electrical conductivity, and facilitated the ion diffusion. The presence of synergistic interactions of MWCNTs and Ni-MOF improved the electrochemical performance for supercapacitors, with an interesting specific capacitance of 900 F/g at 0.5 A/g and stability of 1000 cycles. Another study [117] also explored the use of the Ni_3_(benzene 1, 3, 5-tricarboxylic acid)@polyaniline-rGO nanocomposite (Ni-MOF@PANI-rGO) by employing two-step methods (polymerization and hydrothermal methods). The Ni-MOF@PANI-rGO exhibits high electrical conductivity and excellent electrocatalytic properties, which facilitated electron transport, and interesting power densities of 73.99 Wh/kg and 848.29 W/kg were obtained. It is believed that Ni-MOF@PANI-rGO may play a vital role in bridging the gap between supercapacitors and batteries. The bimetallic Ni-MOFs, GO, rGO, CNTs, g-CN, and their composites with Ni-MOFs have been extensively used for electrochemical sensing and supercapacitors due to their attractive properties. It is understood that bimetallic Ni-MOFs such as NiCo, NiZn, and NiFe-MOFs provide abundant active sites and high porosity with a larger surface area that can boost the electrochemical reactions and enhance the charge storage capability of the modified electrodes. Pristine MOFs suffer from low conductivity and cannot be explored for long-term applications. It was also observed that presence of GO may facilitate electron transfer but the preparation of GO is not environmentally friendly. CNT-based Ni-MOF composites have limited conductivity. It can be seen that Ni-MOF/GNS composites show moderate capacity (737 C/g) compared to other hybrids, indicating room for improvement. The Ni-MOF/MWCNTs@NiCo-LDH structures suffer from complex synthesis and potential cost issues. The Ni-MOF/MWCNTs prepared with mixed-ligand strategies showed decent capacity (900 F/g) and improved ion diffusion but exhibited poor cyclic stability (1000 cycles). Similarly, Ni-MOF@PANI-rGO hybrids exhibited energy density (73.99 Wh/kg) and power density (848.29 W/kg) but the presence of conducting polymers (PANI) may lead to mechanical degradation over long-term cycling.

### 3.3. Ni-MOF/MXene/Polymers

**Figure 8:**
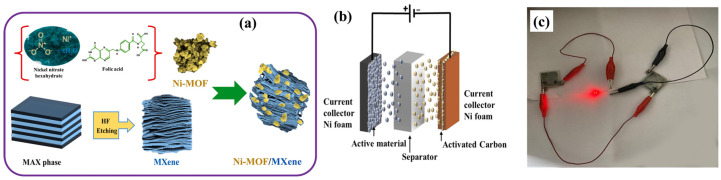
(**a**) Schematic of preparation of Ni-MOF/MXeen composite. (**b**) Schematic of fabricated Ni-MOF/MXene//AC asymmetric supercapacitor device. (**c**) Photograph shows LED light through two Ni-MOF/MXene//AC asymmetric supercapacitors devices. Reprinted with permission from Ref. [119]. 2024, Elsevier.

In another report [118], it was believed that MXene may be promising supporting material with which to enhance the electrochemical energy storage properties of MOFs. In this regard, 3D Ni-MOFs@2D MXene (titanium carbide; Ti_3_C_2_T_x_) was developed for supercapacitor applications. The optimized electrode material (Ni-MOF@MX_2_) shows a specific capacitance of 1160.5 F/g and 736 F/g at 1 A/g and 20 A/g, respectively. In contrast, a bare Ni-MOF-based electrode exhibits a low specific capacitance of 320 F/g at 20 A/g. This reveals that the introduction of MXene enhances the electrochemical properties of the Ni-MOF. The presence of synergistic interactions also enhanced the stability for 1000 cycles at 20 A/g. The Ni-MOF/MXene composite was also formed through solvothermal synthesis-assisted method (Figure 8a) and used for supercapacitor application [119]. Authors have observed that a prepared composite exhibits abundant redox sites and improved electronic conductivity. The specific capacitance of 716.19 F/g was achieved at 1 A/g under optimized conditions. Furthermore, a Ni-MOF/MXene//AC asymmetric supercapacitor device was also fabricated, and its device structure is displayed in Figure 8b. The fabricated Ni-MOF/MXene//AC asymmetric supercapacitor device was able to light an LED, as shown in Figure 8c. This work explored the potential of the Ni-MOF/MXene composite for the development of asymmetric supercapacitors. 

In another study [120], it was observed that the presence of electronegative active sites in MXene materials may facilitate the growth of MOF and form the MXene/Ni-MOF composite through the hydrothermal method. Subsequently, porous nickel phosphate (NPO) was formed using the etching approach. The synthesized MXene/NPO-based electrode exhibits a specific capacitance of 1406 F/g at 1 A/g with a retention capacity of 94.7% after 20,000 cycles. Shalini et al. [121] fabricated a MnNi-MOF/MXene composite for supercapacitor application that delivers a specific capacity of 1028 C/g at 1 A/g. This electrochemical energy storage of the proposed electrode material was attributed to the presence of synergistic effects between MnNi and MXene materials. Gopi et al. [122] prepared a novel Ni-MOF/PANI composite through the hydrothermal method and chemical oxidation polymerization, followed by the sonication approach. The obtained Ni-MOF/PANI composite exhibited a specific capacity of 122 C/g, which is higher than that of pristine Ni-MOF (42 C/g) and PANI (72 C/g). This revealed that the introduction of polymeric materials is of particular importance to enhance the energy storage properties of Ni-MOF. The Ni-MOF/MXene- and Ni-MOF/polymer-based composites demonstrated excellent conductivity and mechanical flexibility for supercapacitors, but the preparation of MXene is not environmentally friendly, whereas polymers may suffers from poor stability for long-term applications.

### 3.4. Ni-MOF/LDH Materials

Wu et al. [123] used novel strategies for the preparation of the NiCo-MOF and LDH nanosheet composite through the solvothermal method. The authors used 1, 4 terephthalic acid and urea for the preparation of the abovementioned composite. It was observed that the hydrolysis of urea released OH-, which serves as the deprotonation of terephthalic acid, and it was also reacted with Ni and Co ions to form the NiCo LDH. The prepared NiCo-MOF@LDH-based electrode exhibits a specific capacitance of 1873.9 F/g at 0.5 A/g with an excellent energy density of 49.8 Wh/kg and power density of 422.4 W/kg. This may be attributed to the larger surface area and abundant active sites generated through the cross-linked nanosheet-like structure. Meng et al. [124] also proposed the fabrication of Ni-MOFs/MWCNTs@NiMn-LDH using the two-step solvothermal method. The specific capacity of 1151 C/g was observed at 1 A/g with decent stability of 5000 cycles at 5 A/g. This shows that the incorporation of LDH and MWCNTs with Ni-MOF facilitated electron transport and improved kinetics for supercapacitors. Although Ni-MOF/LDH materials are novel, next-generation electrode materials for supercapacitor applications still have some limitations, such as structural instability, phase transitions, and the presence of some issues such as uncertainty in interlayer arrangements limiting their applications in electrochemical sensors. Thus, future research may consider such challenges to explore their potential for real-time sensing applications.

### 3.5. Bimetallic and Trimetallic Ni-MOF-Based Materials

Due to their improved physicochemical properties, bimetallic MOFs have been used for supercapacitor applications. In this connection, Guo et al. [125] reported the synthesis of NiCo-MOF by substituting 1, 4 dicarboxybenzene with trimesic acid. Furthermore, NiCo-S was prepared by employing NiCo-MOF as a precursor. The obtained NiCo-S material-based electrode shows higher electrochemical properties for supercapacitors compared to the NiCo-MOF. The optimized conditions revealed a specific capacitance of 1790.4 F. The fabricated asymmetric supercapacitors also demonstrated a specific capacitance of 114.4 F/g with a specific energy of 35.5 Wh/kg at 799.99 W/kg. In further studies, ZnNi-MOF-decorated rGO was fabricated through a one-step solvothermal method [126]. It is clear that rGO sheets may prevent the agglomeration of MOF microspheres, whereas MOF microspheres may act as spacers to inhibit the overlapping of rGO. Thus, the presence of synergistic effects enhanced the electrochemical energy storage properties of the ZnNi-MOF/rGO composite. Under the optimized conditions, a specific capacitance of 1644 F/g was achieved at 1 A/g with reasonably good stability of 3000 cycles. A trimetallic MOF was also proposed as energy storage material for supercapacitors. The sputtering method-based synthesis of molybdenum disulfide (MoS_2_) over NiCoMg-MOF was explored for supercapacitor application [127]. The proposed material exhibited a specific capacitance of 2000 F/g that was 1.8 times higher than that of pristine NiCoMg-MOF. This suggests that the presence of the synergistic interactions between MoS_2_ and NiCoMg-MOF improved specific capacitance. Radhika et al. [128] adopted the solvothermal method for the formation of NiCo-MOF for supercapacitors. It was observed that a specific capacity of 172 C/g was obtained at 2.5 A/g. This may be attributed to the high surface area and decent ion mobility between the electrolyte and electrode. Yue et al. [129] observed that NiCo-MOF can be integrated with aminated Ti_3_C_2_T_x_ MXene, which can exhibit improved electrochemical properties. The NiCo-MOF/Ti_3_C_2_T_x_-NH_2_ shows abundant active sites and significantly contributes to the improved pseudocapacitive performance of supercapacitors. The fabricated electrode can deliver a specific capacitance of 1924 F/g at 0.5 A/g. Another study reported the synthesis of three different types of MOFs using the electrodeposition method [130]. It was observed that the NiCo-MOF-based NF electrode exhibited a specific capacity of 329 C/g at 2 A/g. The coral-like NiCo-MOF was also electrodeposited on the NF electrode [131]. The optimized electrode exhibited a high surface area and a unique hierarchical structure with open channels, providing more active sites and fast ion transfer kinetics. A stability of 10000 cycles was reported under the optimized conditions. In another study [132], various MOF-based electrode materials (CuCoNi-MOF/MoO_3_, CuCoNi-MOF, CuCo-MOF, and Cu-MOF) were fabricated by employing the benign solvothermal method. It was observed that a specific capacitance of 937.5 F/g at 2 mV/s was obtained for the CuCoNi-MOF/MoO_3_-based electrode. Pan et al. [133] introduced laminated stacked NiCo-MOF material, fabricated using the solvothermal method. The modified electrode demonstrated a specific capacitance of 1866 F/g at 1 A/g, which may be attributed to the presence of a lamellar structure and numerous active sites. It is clear from the reported literature that NiCo-MOF-based electrodes display a large number of active sites and enhanced conductivity, which can improve the energy storage properties of the fabricated supercapacitors. The core–shell structure of NiCo-MOF was also fabricated through a post-synthetic approach and optimized conditions, revealing that a specific capacitance of 1176 F/g can be achieved, which is attributed to the porous interconnected sheet-like structure of NiCo-MOF [134]. Marwat et al. [135] fabricated a six-electrode NiCo-MOF/PANI/rGO composite for supercapacitor applications and achieved a specific capacitance of 1007 F/g at 1 A/g. This electrochemical performance may be ascribed to the highly conductive nature of PANI, improved electron transport of rGO, and larger surface area of NiCo-MOF. Furthermore, numerous efforts have been made to fabricate the highly efficient Ni-MOF-based materials for supercapacitor applications, exhibiting decent specific capacitance and reasonable stability [136,137,138,139,140,141,142,143,144,145,146,147,148,149]. It is understood that bimetallic and trimetallic Ni-MOF-based materials exhibit distinct advantages and limitations for supercapacitor applications. NiCo-MOFs shows improved conductivity and synergistic interactions but suffers from structural instability for long-term cyclic stability. It is also clear that the presence of rGO may inhibit or reduce the agglomeration of ZnNi-MOFs and improve ion transport. However, the restacking of rGO may limit its potential for large-scale applications. The trimetallic MOFs are also potential candidates for electrochemical applications but involve the use of multiple steps for their synthesis, which may be time-consuming and affect the reproducibility. The electrochemical performance of the Ni-MOF-based materials for supercapacitor application is summarized in Table 2.

## 4. Conclusions, Challenges, and Future Trends/Perspectives

It is worthy mentioning that Ni-MOF-based materials are promising electrode modifiers for both electrochemical sensors and supercapacitor applications. The excellent properties, such as intrinsic porosity, redox active metal centers, and high specific surface area, of Ni-MOF make it a desirable material for electrochemical applications. The real-time application of pristine Ni-MOF is limited due to its low conductivity, which is supposed to be improved by employing a doping strategy. However, the doping strategy may suffer from low reproducibility. Therefore, numerous composites of Ni-MOF with carbon materials, metal oxides, polymers, MXene, etc., have been fabricated to improve their conductivity and electrochemical properties. As per the reported studies, it was observed that Ni-MOF-based composites with rGO and MXene are promising materials due to their high conductivity and mechanical flexibility. However, some limitations still exists, including harsh conditions for the formation of rGO and MXenes. The stability of Ni-MOF-based composites for long-term applications is also limited. The actual mechanism for the detection of targeted analytes is still not clear, and an in-depth study for the mechanism is required. The mechanistic underlying active site behavior and charge transfer in supercapacitors needs to be studied in details. The utilization of machine learning and computational simulations may be useful for the rational design of electrode materials. It is believed that self-powered and flexible energy storage devices are key future directions, especially for the development of wearable biosensors, portable electronics, and next-generation supercapacitors. The hybrid composites of Ni-MOF with high porosity, conductivity, and surface area and abundant active sites have a promising role in the development of biosensors for healthcare systems, environmental monitoring, and supercapacitors. Wearable biosensors are also a next-generation sensing technology that has several advantages compared to conventional electrochemical sensors. The combined experimental and theoretical studies may also benefit researchers in understanding the real mechanism for sensing and supercapacitor applications. 

## Figures and Tables

**Table 1:** Electrochemical sensing parameters of the reported Ni-MOF-based sensors.

Electrode Modifier	Sensing Analyte	LOD (µM)	Sensitivity	LR (µM)	Technique	Real Sample	Refs
Ni_3_(HITP)_2_/SPCE	AA	1	0.814 μA μM^−1^ cm^−2^	2 to 200	Amp	Human sweat	[41]
Co–Ni-MOFs-1%/GCE	_L_-TRP	0.0087	-	0.01 to 300	DPV	Mice plasma	[43]
Ni-benzimidazole MOFs	Glucose	0.1401	2199.88 mA M^−1^ cm^−2^	0.5 to 2665.5	Amp	-	[45]
Ni-MOF/CeO_2_	Glucose	30	2488 μA mM^−1^ cm^−2^	0.04 mM to 1.2 mM	Amp	Sweat	[50]
Ni-MOF/RGO/GCE	EP	0.018	-	0.05 to 200	LSV	Serum and urine	[51]
Ni-MOF/RGO/GCE	FA	0.016	-	0.01 to 150	LSV	Serum and urine	[51]
Ni-BDC/MWCNTs/GCE	4-CP	0.0165	-	0.1 to 50, 50 to 500	DPV	River water	[53]
β-CD/Ni-MOF/C_60_-GO /GCE	KA	0.058	-	20 to 50	DPV	Broccoli samples	[55]
F_x_GnP-Ni-MOF	BPA	0.000184	-	0.002 to 10	Amp	River and sewage effluent water	[57]
Ni-Fe(PDC)/GR	CBM	0.0032	-	0.05 to 320	DPV	Strawberry and apple juice	[59]
Ni_3_ZnC_0.7_/Ni/GCE	HQ	0.14	-	0.3 to 100	DPV	Yellow River and tap water	[63]
Ni_3_ZnC_0.7_/Ni/GCE	CC	0.21	-	0.5 to 100	DPV	Yellow River and tap water	[63]
GCE/rGO/NiCo-BTC MOFs	CAP	0.235	33.12 μA μM^−1^ cm^−2^	0.1 to 100	DPV	Tap water	[68]
NiCu-MOF-6	Glucose	15	1832 μA mM^−1^ cm^−2^	0.02 to 4.93	Amp	Human serum	[79]

**Table 2:** Electrochemical performance of reported Ni-MOF-based materials for supercapacitor application.

Electrode Modifier	Specific Capacitance	Electrolyte	Current Density (A/g)	Cyclic Stability	Refs
2D Ni-MOF	746 C/g	3 M KOH	1	10,000	[100]
La-doped Ni-MOF	159.9 mA h/g	2 M KOH	1	5000	[102]
Cr-doped Ni-MOF	853 C/g	3 M KOH	1	5000	[103]
Ni-MOF globules	1361 F/g	2 M KOH	0.5	-	[104]
Ni-MOF	2567.23 F/g	6 M KOH	2	5000	[105]
Ni-MOF	565.32 C/g	1 M KOH	1.2	5000	[106]
Ni-MOF nanosheets	1124 F/g	2 M KOH	2	3000	[107]
Ni-MOF	1668.4 F/g	1 M KOH	10	10,000	[108]
V_2_O_5_/Ni-MOF	546 F/g	1 M KOH	1	10,000	[109]
N-Ni MOF	1519 F/g	2 M KOH	1	2000	[110]
Ni-MOF	221 F/g	3 M KOH	1	2000	[111]
ZnCo_2_O_4_@Ni-MOF	1800 F/g	1 M KOH	2	5000	[113]
Ni-MOF/MWCNTs	900 F/g	6 M KOH	0.5	1000	[116]
Ni-MOF@MXene	1160.5 F/g	3 M KOH	1	10,000	[118]
Ni-MOF/MXene	1406 F/g	-	1	20,000	[120]
NiCo-MOF@LDH	1873.9 F/g	2 M KOH	0.5	10,000	[123]
2D NiCo-MOF	1790 F/g	6 M KOH	1	10,000	[125]
Ni/Co-MOF@TCT-NH_2_	1924 F/g	3 M KOH	0.5	10,000	[129]
NiCo-MOFs	1176 F/g	2 M LiOH	5 mV/s	5000	[134]
NiCo-MOF	1070 F/g	1 M KOH	0.5 mA/cm^2^	5000	[136]
NiFe-MOF	15.6 F/cm^2^	1 M KOH	2 mA/cm^2^	5000	[139]
Ni-Co MOF/rGO	1320 F/g	1 M KOH	4 mA/cm^2^	5000	[141]
Ni/Mn-MOF	447.5 F/g	2 M KOH	0.5	10,000	[145]

## Data Availability

No new data were generated. Authors are unable to provide data.

## References

[1] Kumar R., Shafique M.S., Chapa S.O.M., Madou M.J. (2025). Recent Advances in MOF-Based Materials for Biosensing Applications. Sensors.

[2] Dhanabalan K., Perumalsamy M., Sriram G., Murugan N., Shalu, Sadhasivam T., Oh T.H. (2023). Metal–Organic Framework (MOF)-Derived Catalyst for Oxygen Reduction Reaction (ORR) Applications in Fuel Cell Systems: A Review of Current Advancements and Perspectives. Energies.

[3] Maranescu B., Visa A. (2022). Applications of Metal–Organic Frameworks as Drug Delivery Systems. Int. J. Mol. Sci..

[4] Mahmoud E., Ali L., El Sayah A., Alkhatib S.A., Abdulsalam H., Juma M., Al-Muhtaseb A.H. (2019). Implementing Metal–Organic Frameworks for Natural Gas Storage. Crystals.

[5] Ryoo G., Kim S.K., Lee D.K., Kim Y.-J., Han Y.S., Jung K.-H. (2024). Energy Storage Performance of Electrode Materials Derived from Manganese Metal–Organic Frameworks. Nanomaterials.

[6] Rasheed T., Rizwan K., Bilal M., Iqbal H.M.N. (2020). Metal–Organic Framework-Based Engineered Materials—Fundamentals and Applications. Molecules.

[7] Mosca L.P.L., Gapan A.B., Angeles R.A., Lopez E.C.R. (2023). Stability of Metal–Organic Frameworks: Recent Advances and Future Trends. Eng. Proc..

[8] Laeim H., Molahalli V., Prajongthat P., Pattanaporkratana A., Pathak G., Phettong B., Hongkarnjanakul N., Chattham N. (2025). Porosity Tunable Metal–Organic Framework (MOF)-Based Composites for Energy Storage Applications: Recent Progress. Polymers.

[9] Yang Y., Ji W., Yin Y., Wang N., Wu W., Zhang W., Pei S., Liu T., Tao C., Zheng B. (2023). Catalytic Modification of Porous Two-Dimensional Ni-MOFs on Portable Electrochemical Paper-Based Sensors for Glucose and Hydrogen Peroxide Detection. Biosensors.

[10] Zhang L., Zhang M., Yang P., Zhang Y., Fei J., Xie Y. (2023). Electrochemical Behavior of β-Cyclodextrin-Ni-MOF-74/Reduced Graphene Oxide Sensors for the Ultrasensitive Detection of Rutin. Molecules.

[11] Zhang Y., Liu C., Yan R., Lei C. (2024). Bipyridyl Ruthenium-Decorated Ni-MOFs on Carbon Nanotubes for Electrocatalytic Oxidation and Sensing of Glucose. Chemosensors.

[12] Wang Q., Jia Q., Hu P., Ji L. (2023). Tunable Non-Enzymatic Glucose Electrochemical Sensing Based on the Ni/Co Bimetallic MOFs. Molecules.

[13] Dourandish Z., Sheikhshoaie I., Maghsoudi S. (2023). Molybdenum Disulfide/Nickel-Metal Organic Framework Hybrid Nanosheets-Based Disposable Electrochemical Sensor for Determination of 4-Aminophenol in Presence of Acetaminophen. Biosensors.

[14] Sun Z., Wu Z., Zong Y., Li C., Guo W., Guo Y., Zou X. (2024). Construction of Metal–Organic Framework as a Novel Platform for Ratiometric Determination of Cyanide. Biosensors.

[15] Xu X., Zhong Y., Cai S., Lei L., Peng J. (2025). Does Air Pollution Aggravate Health Problems in Low-Income Countries? Verification from Countries Along the Belt and Road. Sustainability.

[16] Yang C., Xue Z., Wen J. (2023). Recent Advances in MOF-Based Materials for Remediation of Heavy Metals and Organic Pollutants: Insights into Performance, Mechanisms, and Future Opportunities. Sustainability.

[17] Alsalme A., Alsaeedi H. (2023). Fabrication of Selective and Sensitive Hydrazine Sensor Using Sol–Gel Synthesized MoSe_2_ as Efficient Electrode Modifier. Crystals.

[18] Zhang Y., Li N., Liu B., Zhang H. (2024). Hydrogen Peroxide and Dopamine Sensors Based on Electrodeposition of Reduced Graphene Oxide/Silver Nanoparticles. Sensors.

[19] Wang T., Xu X., Wang C., Li Z., Li D. (2022). A Novel Highly Sensitive Electrochemical Nitrite Sensor Based on a AuNPs/CS/Ti_3_C_2_ Nanocomposite. Nanomaterials.

[20] Venegas C.J., Bollo S., Sierra-Rosales P. (2023). Carbon-Based Electrochemical (Bio)Sensors for the Detection of Carbendazim: A Review. Micromachines.

[21] Ho C.K., Robinson A., Miller D.R., Davis M.J. (2005). Overview of Sensors and Needs for Environmental Monitoring. Sensors.

[22] Nalakurthi N.V.S.R., Abimbola I., Ahmed T., Anton I., Riaz K., Ibrahim Q., Banerjee A., Tiwari A., Gharbia S. (2024). Challenges and Opportunities in Calibrating Low-Cost Environmental Sensors. Sensors.

[23] Islam M.S., Sazawa K., Sugawara K., Kuramitz H. (2023). Electrochemical Biosensor for Evaluation of Environmental Pollutants Toxicity. Environments.

[24] Bounegru A.V., Dinu Iacob A., Iticescu C., Georgescu P.L. (2025). Electrochemical Sensors and Biosensors for the Detection of Pharmaceutical Contaminants in Natural Waters—A Comprehensive Review. Chemosensors.

[25] Hernandez-Vargas G., Sosa-Hernández J.E., Saldarriaga-Hernandez S., Villalba-Rodríguez A.M., Parra-Saldivar R., Iqbal H.M.N. (2018). Electrochemical Biosensors: A Solution to Pollution Detection with Reference to Environmental Contaminants. Biosensors.

[26] Gupta P.K., Siegenthaler J.R. (2025). Revolutionizing Electrochemical Sensing with Nanomaterial-Modified Boron-Doped Diamond Electrodes. Chemosensors.

[27] Ma C., Wen Y., Qiao Y., Shen K.Z., Yuan H. (2024). A Dopamine Detection Sensor Based on Au-Decorated NiS_2_ and Its Medical Application. Molecules.

[28] Dey K., Santra T.S., Tseng F.G. (2025). Advancements in Glucose Monitoring: From Traditional Methods to Wearable Sensors. Appl. Sci..

[29] Nagal V., Masrat S., Khan M., Alam S., Ahmad A., Alshammari M.B., Bhat K.S., Novikov S.M., Mishra P., Khosla A. (2023). Highly Sensitive Electrochemical Non-Enzymatic Uric Acid Sensor Based on Cobalt Oxide Puffy Balls-like Nanostructure. Biosensors.

[30] Czagany M., Hompoth S., Keshri A.K., Pandit N., Galambos I., Gacsi Z., Baumli P. (2024). Supercapacitors: An Efficient Way for Energy Storage Application. Materials.

[31] Salaheldeen M., Eskander T.N.A., Fathalla M., Zhukova V., Blanco J.M., Gonzalez J., Zhukov A., Abu-Dief A.M. (2025). Empowering the Future: Cutting-Edge Developments in Supercapacitor Technology for Enhanced Energy Storage. Batteries.

[32] Yaseen M., Khattak M.A.K., Humayun M., Usman M., Shah S.S., Bibi S., Hasnain B.S.U., Ahmad S.M., Khan A., Shah N. (2021). A Review of Supercapacitors: Materials Design, Modification, and Applications. Energies.

[33] Subasinghage K., Gunawardane K., Padmawansa N., Kularatna N., Moradian M. (2022). Modern Supercapacitors Technologies and Their Applicability in Mature Electrical Engineering Applications. Energies.

[34] Mehra P., Saxena S., Bhullar S. (2024). A Comprehensive Analysis of Supercapacitors and Their Equivalent Circuits—A Review. World Electr. Veh. J..

[35] Sriram G., Kurkuri M., Oh T.H. (2023). Recent Trends in Highly Porous Structured Carbon Electrodes for Supercapacitor Applications: A Review. Energies.

[36] Argirusis C., Katsanou M.-E., Alizadeh N., Argirusis N., Sourkouni G. (2025). Recent Advances in the Application of MOFs in Supercapacitors. Batteries.

[37] Dutt S., Kumar A., Singh S. (2023). Synthesis of Metal Organic Frameworks (MOFs) and Their Derived Materials for Energy Storage Applications. Clean Technol..

[38] Li Z., Xu J., Ding X., Zhu H., Wu J. (2025). Mechanically Exfoliated Multilayer Graphene-Supported Ni-MOF Parallelogram Nanosheets for Enhanced Supercapacitor Performance. Nanomaterials.

[39] Li Q., Guo H., Xue R., Wang M., Xu M., Yang W., Zhang J., Yang W. (2020). Self-Assembled Mo-Doped Ni-MOF Nanosheets-Based Electrode Material for High Performance Battery–Supercapacitor Hybrid Device. Int. J. Hydrogen Energy.

[40] Li S., Wang Y., Li Y., Xu J., Li T., Zhang T. (2023). In Situ Growth of Ni-MOF Nanorods Array on Ti_3_C_2_T_x_ Nanosheets for Supercapacitive Electrodes. Nanomaterials.

[41] Wang L., Pan L., Han X., Ha M.N., Li K., Yu H., Zhang Q., Li Y., Hou C., Wang H. (2022). A Portable Ascorbic Acid in Sweat Analysis System Based on Highly Crystalline Conductive Nickel-Based Metal–Organic Framework (Ni-MOF). J. Colloid Interface Sci..

[42] Kavya K.V., Muthu D., Pattappan D., Vargheese S., Gokila N., Sivaramkumar M.S., Rajendra Kumar R.T., Haldorai Y. (2022). Palladium Nanoparticles Decorated Ni-MOF Nanocomposite as an Electrochemical Platform for the Selective Detection of Dopamine. Mater. Lett..

[43] Huang W., Chen Y., Wu L., Long M., Lin Z., Su Q., Zheng F., Wu S., Li H., Yu G. (2022). 3D Co-Doped Ni-Based Conductive MOFs Modified Electrochemical Sensor for Highly Sensitive Detection of L-Tryptophan. Talanta.

[44] Wan J., Shen Y., Xu L., Xu R., Zhang J., Sun H., Zhang C., Yin C., Wang X. (2021). Ferrocene-Functionalized Ni(II)-Based Metal–Organic Framework as Electrochemical Sensing Interface for Ratiometric Analysis of Cu^2+^, Pb^2+^ and Cd^2+^. J. Electroanal. Chem..

[45] Wang F., Hu J., Wu X., Yuan G., Su Y., Fan Z., Xue H., Pang H. (2024). Streamlined Synthesis of Superstructure Ni-Benzimidazole MOFs: Glucose Electrochemical Analysis. J. Colloid Interface Sci..

[46] Cao J., Yun J., Zhang N., Wei Y., Yang H., Xu Z. (2021). Bifunctional Ag@Ni-MOF for High Performance Supercapacitor and Glucose Sensor. Synth. Met..

[47] Nie Y., Li Y., Li J., Chen L., Wang X., Chen T., Cai Z. (2024). Incorporated Ferrocene-Derivatives Endow Ni-Based MOF with High-Performance for Electrochemical Detection. Colloids Surf. A Physicochem. Eng. Asp..

[48] Zhang Z., Huang L., Gao F., Zheng Z., Lin Y., Wang S., Wang Q., Wang Q. (2025). A Ratiometric Electrochemical Sensor for Antiepileptic Drug of Carbamazepine Based on Electroactive Ni^2+^-Terephthalic Acid MOF. Talanta.

[49] Ebadi S., Ghanbari K., Zahedi-Tabrizi M. (2025). Development of an Electrochemical Sensor Based on Ni-Bio-MOF and a Molecular Imprinted Polymer for Determination of Diclofenac: Electrochemical and DFT Investigations. RSC Adv..

[50] Atefi A., Moradi S., Salehnia F., Hosseini M. (2025). A Free Enzyme Electrochemical Glucose Sensor Using an Integrated Display to CeO_2_ NPs/Ni-MOF-Based Sensor for Highly Sensitive Determination of Glucose in Sweat. Microchem. J..

[51] Chen A., Tang N., Wei Y., Shi S., Zhou C., He Q., Wang W. (2025). A novel approach to simultaneous and sensitive detection of epinephrine and folic acid with nickel-metal organic framework and reduced graphene oxide based electrochemical sensor. Microchem. J..

[52] Mohseni-Sardari F., Mazloum-Ardakani M., Mohammadian-Sarcheshmeh H., Alizadeh Z., Houshmand S. (2025). Enzyme-free glutamate sensor using peony flower-like structure of nickel metal–organic framework/MWCNTs nanocomposite. Inorg. Chem. Commun..

[53] Ji L., Li F., Jia Q., Yao Y., Zhu X., Li Z., Hu P. (2024). Signal-amplified electrochemical monitoring of 4-chlorophenol in water environments based on Ni-BDC decorated multi-walled carbon nanotubes. J. Environ. Chem. Eng..

[54] Chen A., Tang N., Wei Y., Shi S., Zhou C., He Q., Ding J. (2024). Efficient and environmentally friendly uric acid voltametric sensor based on Ni-MOF and carboxylated multi-walled carbon nanotubes nanocomposites. J. Environ. Chem. Eng..

[55] Tan Q., Chen C., Lin C., Zhang J., Liu S., Zhang J. (2024). Highly sensitive detection of kaempferol using electrochemical sensors based on 3D-ordered mesh interconnect C60-GO, Ni-MOF, and β-cyclodextrin. Microchem. J..

[56] Dong S., Niu H., Sun L., Zhang S., Wu D., Yang Z., Xiang M. (2022). Highly dense Ni-MOF nanoflake arrays supported on conductive graphene/carbon fiber substrate as flexible microelectrode for electrochemical sensing of glucose. J. Electroanal. Chem..

[57] Arul P., Huang S.-T., Gowthaman N.S.K., Mani G., Jeromiyas N., Shankar S., John S.A. (2021). Electrocatalyst based on Ni-MOF intercalated with amino acid-functionalized graphene nanoplatelets for the determination of endocrine disruptor bisphenol A. Anal. Chim. Acta.

[58] Yan Z., Wu Y.-P., Hu Y., Li S., Yin Y.-M., Wu X.-Q., Li D.-S. (2025). Ti_3_C_2_@Ni-MOF heterostructure as efficient bifunctional sensor for highly sensitive detection of adenine and ascorbic acid. Mater. Lett..

[59] Dey B., Kushwaha K.S., Ullah I., Kamal T., Khan S., Ahmad M.W., Hossain S.S., Choudhury A. (2025). Highly sensitive and selective electrochemical sensor for carbendazim detection in fruit juice using novel bimetallic metal–organic framework anchored graphite rod electrode. Inorg. Chem. Commun..

[60] Dong Y., Li J., Zeng H., Li T., Hu D., Li Z., Fu Q., Bateer B. (2025). Construction of bimetallic MOF derived nickel–cobalt selenide on carbon cloth as integrated electrode for acetaminophen sensing. Microchem. J..

[61] Zaimbashi R., Salarizadeh N., Askari M.B. (2025). Electrochemical sensor based on Ni-Co-MOF/MWCNTs nanocomposite and benzoyl ferrocene for determination of L-cysteine in the presence of tryptophan. Inorg. Chem. Commun..

[62] Zhou Y., Zhang L., Li J., Hu M., Zhuang G., Xiong X. (2025). Novel self-supporting CoNi-MOF with triangular cone geometry array structure for highly sensitive electrochemical sensing of fructose in beverages. Colloids Surf. A Physicochem. Eng. Asp..

[63] Liao Y., Lin L., Liu J., Zhang X., Li X. (2024). Ultrasensitive simultaneous determination of hydroquinone and catechol by Ni_3_ZnC_0.7_/Ni porous composites derived from Ni/Zn-MOF. J. Electroanal. Chem..

[64] Zou J., Zou J., Li L., Chen H., Liu S., Gao Y., Huang X., Wang L., Lu L. (2024). Enhanced electrocatalytic activity in MOFs-derived 3D hollow NiCo-LDH nanocages decorated porous biochar for simultaneously ultra-sensitive electrochemical sensing of Cu^2+^ and Hg^2+^. Talanta.

[65] Wang C., Zhang Y., Liu Y., Zeng X., Jin C., Huo D., Hou J., Hou C. (2024). A wearable flexible electrochemical biosensor with CuNi-MOF@rGO modification for simultaneous detection of uric acid and dopamine in sweat. Anal. Chim. Acta.

[66] Li Y., Wang W., Yue W., Lei Q., Zhao Z., Sun Y., Xu H., Zhang W., Chen L., Kim J.K. (2024). Construction of highly sensitive electrochemical immunosensor based on Au and Co_3_O_4_ nanoparticles functionalized Ni/Co bimetal conductive MOF for quantitative detection of HBsAg. Chem. Eng. J..

[67] Ahmad M.M., Roushani M., Farokhi S. (2024). Ni–P nanosheets derived from a metal–organic framework containing triptycene ligand: A high-performance electrochemical sensor for glucose determination. Microchem. J..

[68] Han S., Zhang M., Yang J., Zhang N., Yan R., Wang L., Gao L., Zhang Z. (2024). A highly sensitive electrochemical sensor for the detection of chloramphenicol based on Ni/Co bimetallic metal–organic frameworks/reduced graphene oxide composites. J. Electroanal. Chem..

[69] Gao H., Chai J., Jin C., Tian M. (2024). Molecularly imprinted electrochemical sensor based on Co–Ni-MOF/RGO nanocomposites for sensitive detection of hippuric acid. Anal. Chim. Acta.

[70] Liu J., Sun G., Sun W., Zha X., Wang N., Wang Y. (2024). Portable electrochemical sensor for adrenaline detection using CoNi-MOF-based CS-PAM hydrogel. J. Colloid Interface Sci..

[71] Sun H.-N., Wang M., Liu X.-Y., Zhao L.-X., Li S.-S. (2024). Electrochemical ratiometric biosensor based on 2D flower-like Co/Ni MOF and sea urchin-like PdCuNi for accurate quantification of alpha-fetoprotein. Chem. Eng. J..

[72] Shu H., Lai T., Yang Z., Xiao X., Chen X., Wang Y. (2023). High sensitivity electrochemical detection of ultra-trace imidacloprid in fruits and vegetables using a Fe-rich FeCoNi-MOF. Food Chem..

[73] Wang Y., Zhai H., Guo Q., Zhang Y., Sun X., Guo Y., Yang Q., Zhang Y. (2023). Shared hairpin structure electrochemical aptasensor based on ZrO_2_@Ni/Co-MOFs@AuNPs for dual-target detection of Cd^2^^+^ and *S. aureus*. Sens. Actuators B Chem..

[74] Shu Y., Su T., Lu Q., Shang Z., Feng J., Jin D., Zhu A., Xu Q., Hu X. (2022). Paper-based electrochemical immunosensor device via Ni–Co MOF nanosheet as a peroxidase mimic for the label-free detection of alpha-fetoprotein. Sens. Actuators B Chem..

[75] Wang W., Zhao Z., Lei Q., Sun Y., Zhang W., Zhuiykov S., Zhang W., Hu J. (2022). Constructing and electrochemical performance of AuNPs decorated MIL-53 (Fe, Ni) MOFs–derived nanostructures for highly sensitive hydrazine detection. Appl. Surf. Sci..

[76] Yin M., Zhang L., Wei X., Sun Y., Qi S., Chen Y., Tian X., Qiu J., Xu D. (2022). Spongy Co/Ni-Bio-MOF-Based Electrochemical Aptasensor for Detection of Kanamycin Based on Coral-Like ZrO_2_@Au as an Amplification Platform. J. Electroanal. Chem..

[77] Tang J., Hu T., Li N., Zhu Y., Li J., Zheng S., Guo J. (2022). Ag Doped Co/Ni Bimetallic Organic Framework for Determination of Luteolin. Microchem. J..

[78] Umesh N.M., Jesila J.A., Wang S.-F., Vishnu N., Yang Y.-J. (2021). Novel Voltammetric Detection of Norfloxacin in Urine and Blood Serum Using a Flexible Ni Foam Based Ni-Co-MOF Ultrathin Nanosheets Derived from Ni-Co-LDH. Microchem. J..

[79] Pan W., Zheng Z., Wu X., Gao J., Liu Y., Yuan Q., Gan W. (2021). Facile Synthesis of 2D/3D Hierarchical NiCu Bimetallic MOF for Non-Enzymatic Glucose Sensor. Microchem. J..

[80] Song Y., Xu M., Liu X., Li Z., Wang C., Jia Q., Zhang Z., Du M. (2021). A Label-Free Enrofloxacin Electrochemical Aptasensor Constructed by a Semiconducting CoNi-Based Metal–Organic Framework (MOF). Electrochim. Acta.

[81] Xu Z., Wang Q., Zhangsun H., Zhao S., Zhao Y., Wang L. (2021). Carbon Cloth-Supported Nanorod-Like Conductive Ni/Co Bimetal MOF: A Stable and High-Performance Enzyme-Free Electrochemical Sensor for Determination of Glucose in Serum and Beverage. Food Chem..

[82] Li S., Xie X., Zhang N., Li C., Li Y., Jiang M., Huang P., Jin H. (2025). Nonlinear Device Strategy of Non-Enzyme Glucose Sensor via Amorphous Ni MOF and Crystalline Ni_3_(PO_4_)_2_ Composite. Microchem. J..

[83] Gao P., Hussain M.Z., Gryc D., Mukherjee S., Zhou Z., Li W., Jentys A., Elsner M., Fischer R.A. (2025). Enhanced Electrochemical Activity by MOF Superstructure Derived Ni_2_P@C for Ultrasensitive Sensing of Bisphenol A. Biosens. Bioelectron..

[84] Afruz A., Amiri M., Kaffash-Jamshid M., Bezaatpour A., Bottke P., Wark M. (2025). Dual-Function Nickel Bio-MOF as a Non-Enzymatic Glucose Sensor and Efficient Supercapacitor. Electrochim. Acta.

[85] Xiong Q., Chen S., Pei L., Liu J., Yang Y., Lu M., Song Y. (2025). High-Selective Dopamine Electrochemical Sensor Based on Yolk-Shell Structural Composites Derived from Ni-MOF@COFTAPB-DVA. Microchem. J..

[86] Zhu Z., Lv T., Wang Y., Nan S., Haotian R., Yang Q., Liang A., Luo A. (2025). A Novel Molecularly Imprinted Electrochemical Biosensor Based on Ni_3_(HITP)_2_-MOF and a Novel Anti-Fouling Material for the Direct Detection of Glucose in Whole Blood. Sens. Actuators B Chem..

[87] Rasheed S., Ikram M., Fatima B., Alomayri T., Hussain D. (2025). Facile Synthesis of MOF-Derived Graphitic Carbon-Decorated NiVO_4_ as an Ultra-Sensitive Electrochemical Sensor for Non-Enzymatic Detection of Sarcosine. J. Alloys Compd..

[88] Yang C., Li S., Liao C., Du C., Yao H., Li Y., Zhang Y., Stachewicz U., Liu Y. (2026). A Wearable 3D Nanostructured Ni-MOF Electrochemical Sensor Integrated with Janus Fabric for Sweat Collecting and Nutrients Detecting. Talanta.

[89] Xiang M., Wu J., Lu T., Lin W., Quan M., Ye H., Dong S., Yang Z. (2024). Ni-MOFs Grown on Carbonized Loofah Sponge for Electrochemical Glucose Detection: Effects of Different Carboxylic Acid Ligands and Reaction Temperatures on Electrochemical Performance. J. Taiwan Inst. Chem. Eng..

[90] Han S., Sun R., Zhao L., Yan C., Chu H. (2023). Molecularly Imprinted Electrochemical Sensor Based on Synergistic Interaction of Honeycomb-Like Ni-MOF Decorated with AgNPs and N-GQDs for Ultra-Sensitive Detection of Olaquindox in Animal-Origin Food. Food Chem..

[91] Lu Z., Wei K., Ma H., Xiong Q., Li Y., Sun M., Wang X., Wang Y., Wu C., Su G. (2023). Nanoarchitectonics of On–Off Ratiometric Signal Amplified Electrochemical Sensor for Chlorpromazine with Molecularly Imprinted Polymer Based on Ni-MOF/Fe-MOF-5 Hybrid Au Nanoparticles. Sep. Purif. Technol..

[92] Chen C., Ren J., Zhao P., Zhang J., Hu Y., Fei J. (2023). A Novel Dopamine Electrochemical Sensor Based on a β-Cyclodextrin/Ni-MOF/Glassy Carbon Electrode. Microchem. J..

[93] Wei P., Wang S., Wang W., Niu Z., Rodas-Gonzalez A., Li K., Li L., Yang Q. (2022). CoNi Bimetallic Metal–Organic Framework and Gold Nanoparticles-Based Aptamer Electrochemical Sensor for Enrofloxacin Detection. Appl. Surf. Sci..

[94] Xiao L., Yang K., Duan J., Zheng S., Jiang J. (2022). The Nickel Phosphate Rods Derived from Ni-MOF with Enhanced Electrochemical Activity for Non-Enzymatic Glucose Sensing. Talanta.

[95] Pang L., Jia X., Wang P., Wang Y., Yang Y., Liu H. (2022). Bimetallic Synergy Boost TCPP(Ni)-Co MOF as the High-Performance Electrochemical Sensor for Enhanced Detection of Trace Theophylline. Microchem. J..

[96] Guo L., Hao L., Zhang Y., Yang X., Wang Q., Wang Z., Wang C. (2021). Metal–Organic Framework Precursors Derived Ni-Doping Porous Carbon Spheres for Sensitive Electrochemical Detection of Acetaminophen. Talanta.

[97] Zhang X., Wang R., Wei Y., Pei X., Zhou Z., Zhang J., Zhang R., Zhang D. (2021). MOF-Derived Porous NiO Nanorod and Microflower Structures with Enhanced Non-Enzymatic Glucose Electrochemical Sensing Performance. Int. J. Electrochem. Sci..

[98] Li G., Liu S., Liu D., Zhang N. (2021). MOF-Derived Porous Nanostructured Ni_2_P/C Material with Highly Sensitive Electrochemical Sensor for Uric Acid. Inorg. Chem. Commun..

[99] Yang W., Guo H., Fan T., Zhao X., Zhang L., Guan Q., Wu N., Cao Y., Yang W. (2021). MoS_2_/Ni(OH)_2_ Composites Derived from In Situ Grown Ni-MOF Coating MoS_2_ as Electrode Materials for Supercapacitor and Electrochemical Sensor. Colloids Surf. A Physicochem. Eng. Asp..

[100] Xia K., Yi F., Zheng L., Gao A., Shu D., Ling J. (2023). 2D Coordination Unsaturated Ni-MOFs Hierarchical Nanosheets with Internal Electric Fields for High-Performance Hybrid Supercapacitors. J. Electroanal. Chem..

[101] Bhoite A.A., Patil K.V., Redekar R.S., Patil P.S., Sawant V.A., Tarwal N.L. (2023). Solvothermal synthesis of binder-free Ni-MOF thin films for supercapacitor electrodes. J. Solid State Chem..

[102] Ding Y., Yan Z., Wang G., Sang H., Li W., Xu Z. (2023). One-pot preparation of La-doped Ni-MOF nanospheres for efficient hybrid supercapacitor electrode material. Mater. Chem. Phys..

[103] Sun X., Chen J., Kong W., Yu Q., Hu C., Long Y., Dai Y., Gong J., Pu L., Zhang H. (2023). Cr-doped Ni-MOF nanosheet array structure anchored on nickel foam with specific orientation for high performance supercapacitors. Electrochim. Acta.

[104] Chaturvedi G., Jaiswal R., Ilangovan S.A., Sujatha S., Ajeesh K.S., Tatiparti S.S.S.V. (2023). A systematic approach for selecting suitable morphologies for supercapacitor applications through morphological stability map—A case of Ni-MOF. Ceram. Int..

[105] Liu R., Shang M.Y., Liu C., Hao Y., Yang F., Shi J.Y., Chen Y., Wang Y.F., Feng J.Q., Yang P.F. (2024). Effect of hydrochloric acid on the properties of Ni-MOF nanostructures as supercapacitor electrode materials. Chem. Phys. Lett..

[106] Khan J., Ahmed A., Saleem M.I., Al-Kahtani A.A. (2024). Benzene-1,4-dicarboxylic acid-based Ni-MOF for efficient battery–supercapacitor hybrids: Electrochemical behavior and mechanistic insights. J. Energy Storage.

[107] Khokhar S., Chand P., Anand H. (2024). Solvent-regulated fabrication of Ni-MOF-based asymmetric supercapacitor device. Inorg. Chem. Commun..

[108] Raje P.G., Gurav S.R., Waikar M.R., Chodankar G.R., Shembade U.V., Moholkar A.V., Dongale T.D., Sonkawade R.G. (2024). Exploring the role of metal concentrations on the chemically synthesized Ni-MOF nanostructures for asymmetric supercapacitor. J. Energy Storage.

[109] Kashif M., Thangarasu S., Murugan N., Magdum S.S., Kim Y.A., Kurkuri M., Oh T.-H. (2024). Interatomic interaction of 2D crumpled V_2_O_5_ nanosheets layered with Ni-MOF as a bifunctional electrocatalyst for overall water splitting and supercapacitor applications. J. Energy Storage.

[110] Rajamany R., Prakash S., Ismail Y.A. (2025). Polyvinylpyrrolidone-assisted synthesis of Ni-MOF: Enhanced supercapacitive performance through morphology control. Next Mater..

[111] Sundarraj N.K., Pandiyan J.Q.S., Al Souwaileh A., Wu J.J., Anandan S. (2025). Sonochemical synthesis of Ni-MOF using 2-methylimidazole as an organic linker: Pushing the boundaries of energy storage. Electrochim. Acta.

[112] Zhang S., Bi J., Zhan Q., Liu N., Zhang X., Wang Z., Han Y. (2025). Fabrication of a vanadium-doped Ni-MOF with a hydrangea-like morphology as an electrode for high-performance supercapacitors. Chem. Commun..

[113] Bhanuse G.B., Kumar S., Chien C.-W., Fu Y.-P. (2025). Development of heterostructured ZnCo_2_O_4_@Ni-MOF electrode for the asymmetric supercapacitor and electrocatalytic oxygen evolution reaction applications. Electrochim. Acta.

[114] Wang J.-W., Meng T.-L., Ma Y.-X., Lei L., Li J., Ran F. (2023). Fabrication of graphite nanosheets decorated with Ni-MOFs for high-performance supercapacitor electrode materials. Diam. Relat. Mater..

[115] Meng T.-L., Ma Y.-X., Wang J.-W., Li J., Lei L., Wang F., Ran F. (2024). Multicomponent hierarchical Ni-MOFs/MWCNTs@Ni/Co-LDH nanohybrid as advanced electrode material for supercapacitor. J. Phys. Chem. Solids.

[116] Yazdani S., Lashkenari M.S., Mehri F. (2024). Design of a novel mixed-ligand Ni-MOF/MWCNT nanocomposite to enhance the electrochemical performance of supercapacitors. Synth. Met..

[117] Amirabad T.N., Ensafi A.A., Mousaabadi K.Z., Rezaei B., Demir M. (2023). Binder-free engineering design of Ni-MOF ultrathin sheet-like grown on PANI@GO decorated nickel foam as an electrode for hydrogen evolution reaction and asymmetric supercapacitor. Int. J. Hydrogen Energy.

[118] Ji Y., Li W., You Y., Xu G. (2024). In situ synthesis of M (Fe, Cu, Co and Ni)-MOF@MXene composites for enhanced specific capacitance and cyclic stability in supercapacitor electrodes. Chem. Eng. J..

[119] Shivade D.S., Kurade A.N., Bhosale R.K., Kundale S.S., Shelake A.R., Patil A.D., Waifalkar P.P., Kamat R.K., Teli A.M., Dongale T.D. (2024). Folic acid-assisted in situ solvothermal synthesis of Ni-MOF/MXene composite for high-performance supercapacitors. J. Energy Storage.

[120] Li J., Qiang X., Jia B., Wang L., Wu X. (2025). Etching and surface self-assembly of Ni-MOF/MXene hybrids for excellent flexible pseudocapacitance. Appl. Surf. Sci..

[121] Shalini S.S., Fu Y.-P., Bose A.C. (2024). Synergetic interplay of MnNi-MOF composite with 2D MXene for improved supercapacitor application. Chem. Eng. J..

[122] Gopi R.R., Ebenezer T., Prabu H.J., Johnson I., Galeb W., Raja M.D., Sundaram S.J., Kennedy Arockiasamy J.S., Sahayaraj A.F. (2024). Synthesis and investigation of charge storage characteristics in Ni-MOF/PANI composite as an active electrode material for supercapacitor. Electrochim. Acta.

[123] Wu S., Cai D., Tian Z., Guo L., Wang Y. (2024). One-step synthesis of NiCo-MOF@LDH hybrid nanosheets for high-performance supercapacitor. J. Energy Storage.

[124] Meng T.-L., Wang J.-W., Ma Y.-X., Chen X.-Q., Lei L., Li J., Ran F. (2024). Fabrication of a novel nanohybrid via the introduction of Ni/Mn-LDH into Ni-MOFs/MWCNTs for high-performance electrochemical supercapacitor. Diam. Relat. Mater..

[125] Guo H., Zhang H., Wu N., Pan Z., Li C., Chen Y., Cao Y., Yang W. (2023). Trimesic acid-modified 2D Ni-Co-MOF for high-capacity supercapacitors. J. Alloys Compd..

[126] Jin J., Wang N., Wang Y., Wang Y., Sun T. (2023). Synergistic effect of bimetal (Zn/Ni)–organic framework/reduced graphene oxide for high-performance supercapacitor. Appl. Surf. Sci..

[127] Khan M.F., Marwat M.A., Abdullah, Shah S.S., Karim M.R.A., Aziz M.A., Din Z.U., Ali S., Adam K.M. (2023). Novel MoS_2_-sputtered NiCoMg MOFs for high-performance hybrid supercapacitor applications. Sep. Purif. Technol..

[128] Radhika M.G., Srilakshmi R., Tejashree V., Venkatesh K., Kamath M.K.S., Nagaraju K. (2023). A new strategy for the morphology-controlled synthesis of Ni/Co MOFs for high-performance asymmetric supercapacitors. J. Energy Storage.

[129] Yue L., Chen L., Wang X., Lu D., Zhou W., Shen D., Yang Q., Xiao S., Li Y. (2023). Ni/Co-MOF@aminated MXene hierarchical electrodes for high-stability supercapacitors. Chem. Eng. J..

[130] Salehi S., Ehsani M.H., Aghazadeh M. (2023). Direct electrosynthesis of Ni-, Co-, and Ni,Co-MOF onto porous support for high-performance supercapacitors. J. Alloys Compd..

[131] Zhou Y., Zheng Z., Yu Y., Han Y. (2023). Electrodeposited coral-like bimetallic NiCo-MOFs on Ni foam as binder-free electrodes for high performance all solid-state asymmetric supercapacitors. Electrochim. Acta.

[132] Fatima S., Shabbir H., Sharif R., Fahad H.M., Yang J., Shaheen F., Wahab R., Akbar S., Perumal V. (2024). A novel binary composite of CuCoNi-MOF/MoO_3_ with exceptional capacitance as electrode material for supercapacitors. J. Energy Storage.

[133] Pan Q., Yang M., Song F., Xiong Z., He X. (2024). Preparation of layered NiCo-MOF nanosheets for high-performance asymmetric supercapacitor electrode material. Vacuum.

[134] Gurav S.R., Shembade U.V., Patil A.V., Waikar M.R., Sonkawade A.R., Vhatkar R.S., Moholkar A.V., Sonkawade R.G. (2024). Time and cost efficient post-synthesized core-shell NiCo-MOFs electrode for solid-state supercapacitors. Mater. Today Sustain..

[135] Marwat M.A., Ishfaq S., Adam K.M., Tahir B., Shaikh M.H., Khan M.F., Karim M.R.A., Din Z.U., Abdullah S., Ghazanfar E. (2024). Enhancing supercapacitor performance of Ni–Co–Mn metal–organic frameworks by compositing it with polyaniline and reduced graphene oxide. RSC Adv..

[136] Bhoite A.A., Sawant V.A., Tarwal N.L. (2024). Solvothermal synthesis of Ni/Co-based metal-organic framework with nanosheets-like structure for high-performance supercapacitor. Colloids Surf. A Physicochem. Eng. Asp..

[137] Liang J., Qin S., Luo S., Wang Y., Feng J., Liu K., Liao S., Xu Z., Li J. (2024). Epitaxially growing multilayer CoNi-MOFs nanosheets on activated carbon cloth for high-performance asymmetric supercapacitors. J. Power Sources.

[138] Sun L.-J., Zhang X.-Y., Bai C., Guo H.-L., Han C.-M., Zhang Y.-F., Hu H.-M. (2024). Cobalt/nickel 2D MOF nanosheets with a bithiophene-tetraterpyridyl derivative ligand for high-performance supercapacitors through boosting pseudocapacitance. J. Energy Storage.

[139] Dong W., Liu Z., Sun H., Shi Z., Xu J. (2024). Ultrathin defect-rich nanosheets of NiFe-MOF with high specific capacitance and stability for supercapacitor. Mater. Today Chem..

[140] Pallavolu M.R., Banerjee A.N., Roy N., Merum D., Nallapureddy J., Joo S.W. (2024). Faradaic-dominated intercalation pseudocapacitance in bimetallic ultrathin NiMn-MOF nanosheet electrodes for high-performance asymmetric supercapacitors. Chem. Eng. J..

[141] Salunkhe A.D., Pawar P.S., Pagare P.K., Torane A.P. (2024). Facile solvothermal synthesis of Ni-Co MOF/rGO nanoflakes for high-performance asymmetric supercapacitor. Electrochim. Acta.

[142] Wu H., Li S., Liu Y., Mu Q., Shi Y. (2024). Dual metal MOF derived Co-Ni/rGO as cathode material with synergetic effect for an asymmetric supercapacitor with enhanced performances. J. Energy Storage.

[143] Septiani N.L.W., Wustoni S., Failamani F., Wehbe N., Eguchi M., Nara H., Inal S., Suendo V., Yuliarto B. (2024). Revealing the effect of cobalt content and ligand exchange in the bimetallic Ni–Co MOF for stable supercapacitors with high energy density. J. Power Sources.

[144] Feng J., Luo S., Xu P., Liang J., Qin S., Li J. (2025). Understanding the phase structure evolution and charge storage mechanism of FeCoNi-MOFs as electrodes for asymmetric supercapacitors. J. Colloid Interface Sci..

[145] Li B., Zhao R., Yue H., Zhang Y., Lu S., Shang W., Zhang Z., Wen Y. (2025). In situ electrodeposition construction of a Ni/Mn bimetallic organic framework material and its electrochemical performance in flexible supercapacitors. J. Alloys Compd..

[146] Shabbir H., Fahad H.M., Sharif R., Butt A., Fatima S., Shaheen F., Jose R., Wahab R., Perumal V., Akbar S. (2025). Synergistic effect of 3D porous tri-metallic MOF based electrode materials for highly stable asymmetric supercapacitors. Mater. Sci. Semicond. Process..

[147] Arshad I., Marwat M.A., Nawaz H., Hannan M., Abdullah S.M., Humayun M., Bououdina M., Hamayun U., Khan M.Z., Arif A. (2025). Enhanced electrochemical performance of NiCoMn MOFs with Ag-citrate/SWCNT nanocomposites for high-energy supercapacitors. Diam. Relat. Mater..

[148] Li J., Du R., Wang R., Feng J., Luo S. (2025). Rational construction of multilayer NiCo-MOF@MnO_2_ heterostructures with optimized charge storage behavior for asymmetric supercapacitors. Appl. Surf. Sci..

[149] Deyab M.A., Mohsen Q., El-Shamy O.A.A. (2025). Designing novel Bi-metallic MOFs with optimized Ni and Co ions ratios for enhanced supercapacitor performance. J. Energy Storage.

